# Identification of Genes Related to Growth and Lipid Deposition from Transcriptome Profiles of Pig Muscle Tissue

**DOI:** 10.1371/journal.pone.0141138

**Published:** 2015-10-27

**Authors:** Zhixiu Wang, Qinggang Li, Yangzom Chamba, Bo Zhang, Peng Shang, Hao Zhang, Changxin Wu

**Affiliations:** 1 National Engineering Laboratory For Animal Breeding, China Agricultural University, Beijing, People’s Republic of China; 2 Institute of Animal Sciences and Veterinary Medicine, Anhui Academy of Agricultural Sciences, Hefei, People’s Republic of China; 3 College of Animal Science, Tibet Agriculture and Animal Husbandry University, Linzhi, People’s Republic of China; Huazhong University of Science and Technology, CHINA

## Abstract

Transcriptome profiles established using high-throughput sequencing can be effectively used for screening genome-wide differentially expressed genes (DEGs). RNA sequences (from RNA-seq) and microRNA sequences (from miRNA-seq) from the tissues of *longissimus dorsi* muscle of two indigenous Chinese pig breeds (Diannan Small-ear pig [DSP] and Tibetan pig [TP]) and two introduced pig breeds (Landrace [LL] and Yorkshire [YY]) were examined using HiSeq 2000 to identify and compare the differential expression of functional genes related to muscle growth and lipid deposition. We obtained 27.18 G clean data through the RNA-seq and detected that 18,208 genes were positively expressed and 14,633 of them were co-expressed in the muscle tissues of the four samples. In all, 315 DEGs were found between the Chinese pig group and the introduced pig group, 240 of which were enriched with functional annotations from the David database and significantly enriched in 27 Gene Ontology (GO) terms that were mainly associated with muscle fiber contraction, cadmium ion binding, response to organic substance and contractile fiber part. Based on functional annotation, we identified 85 DEGs related to growth traits that were mainly involved in muscle tissue development, muscle system process, regulation of cell development, and growth factor binding, and 27 DEGs related to lipid deposition that were mainly involved in lipid metabolic process and fatty acid biosynthetic process. With miRNA-seq, we obtained 23.78 M reads and 320 positively expressed miRNAs from muscle tissues, including 271 known pig miRNAs and 49 novel miRNAs. In those 271 known miRNAs, 20 were higher and 10 lower expressed in DSP-TP than in LL-YY. The target genes of the 30 miRNAs were mainly participated in MAPK, GnRH, insulin and Calcium signaling pathway and others involved cell development, growth and proliferation, etc. Combining the DEGs and the differentially expressed (DE) miRNAs, we drafted a network of 46 genes and 18 miRNAs for regulating muscle growth and a network of 15 genes and 16 miRNAs for regulating lipid deposition. We identified that *CAV2*, *MYOZ2*, *FRZB*, miR-29b, miR-122, miR-145-5p and miR-let-7c, etc, were key genes or miRNAs regulating muscle growth, and *FASN*, *SCD*, *ADORA1*, miR-4332, miR-182, miR-92b-3p, miR-let-7a and miR-let-7e, etc, were key genes or miRNAs regulating lipid deposition. The quantitative expressions of eight DEGs and seven DE miRNAs measured with real-time PCR certified that the results of differential expression genes or miRNAs were reliable. Thus, 18,208 genes and 320 miRNAs were positively expressed in porcine *longissimus dorsi* muscle. We obtained 85 genes and 18 miRNAs related to muscle growth and 27 genes and 16 miRNAs related to lipid deposition, which provided new insights into molecular mechanism of the economical traits in pig.

## Introduction

Growth rate, meat quality, and meat flavor are the main economic traits in pig production that can influence human consumption of meat products. Fatness traits such as back fat thickness and intramuscular fat content (IMF), which have positive correlations with meat tenderness, juiciness, and taste[1], are economically important in pig breeding because these can influence meat quality and carcass composition.

Chinese indigenous pig breeds, the Diannan Small-ear pig (DSP) and the Tibetan pig (TP), have lower growth rate, more fat deposition, and better meat quality than the introduced pig breeds, such as the Landrace (LL) and Yorkshire (YY), which are lean-type pig breeds characterized by a fast growth rate and high lean meat content[2]. Specifically, the TP and the DSP are mini-pig breeds that have growth traits distinctive from the introduced breeds. Different patterns of muscle growth among these breeds make them a good model for identifying the functional genes responsible for the molecular mechanisms that control the aforementioned economical traits.

The candidate gene approach is currently the main strategy for studying functions of a single gene. However, growth and meat quality traits are complex quantitative traits that are controlled by many interacting genes. Massive parallel sequencing accomplished with the use of a next-generation sequencing (NGS) has the potential for the elucidation of global gene expression. Novel and low-abundance transcripts can be efficiently identified via transcriptome profiling. RNA sequencing (RNA-Seq)is rapidly developing for transcriptome profiling.

Several RNA-seq studies have been reported in different tissues of pigs, such as the skeletal muscle[3–8], gonad[9], liver[10, 11], adipose tissue[12, 13], endometrium[14],which provided better understanding of the mRNA transcriptome in pig. MicroRNAs (miRNAs) play an important role in post-transcriptional regulation of metabolism in cells, such as β-cells, muscle cells, and adipocytes[15]. Global miRNA abundance has been assessed by microarray in skeletal muscle to evaluate the roles of miRNAs in pig development and meat production[16–18].miRNA-seq also provides valuable insights into the miRNA transcriptome, especially into those miRNAs insufficiently detected by microarray analysis. Until now, the porcine miRNA transcriptome has been investigated by NGS in intestine[19], pre- and postnatal piglets[20], developing brain[21], and skeletal muscle[22].

In this study, we use the NGS to generate transcriptome profiles of muscle tissue in the Chinese indigenous pig breeds (DSP and TP) and the introduced pig breeds (LL and YY)to comparatively study the genome-wide expression and gene-miRNA interation between the breeds with extremely distinct phenotypeswhich allowed us to identify the functional genes and the regulation networks that control muscle growth and fat deposition in pigs.Clarifying the complexity of the transcriptome of the pig is beneficial to an understanding of the complex traits that are also associated with humans, such as growth, obesity and metabolism, because pigs share pathological, physiological, and genomic featureswith humans [23, 24].

## Materials and Methods

### Animals and samples

Two indigenous pig breeds, DSP and TP, and two introduced pig breeds, LL and YY, were raised in Beijing Shunyi Pig Breeding Farm with standard rations and water which are not involved in tissues of protection of wildlife. The four pig breeds are all domestic pig and our study are not involved in tissues of endangered or protected wildlife. Eight individuals from each group were slaughtered at 6 months of age with humanly normal procedure. The pigs were treated by electric shock to death and immediately hoisted for bleeding, and then dehaired and dissected the carcass. The *longissimus dorsi* (LD) muscle tissues at the 12th rib were collected and snap-frozen in liquid nitrogen for extraction of the total RNA and miRNA, and LD samples (weighing about 50 g) were collected for measurement of IMF content. Animal care and all experimentation were conducted in accordance with the guidelines approved by the State Key Laboratory for Agro-biotechnology of China Agricultural University (Approval number: XK257).

### Determination of IMF content

The IMF content of LD muscle samples was determined after extraction of crude fat using Soxhlet Extraction (SZF-06A, Shanghai Xinjia Electronic Co., Ltd., Shanghai, China) with petroleum ether (boiling temperature range: 60°C to 90°C). The extraction followed a previously described method[25]that provides highly accurate measurements with a considerably shorter extraction time compared with other methods. Three replications were performed for each sample.

### RNA isolation from LD muscle samples

Total RNA for mRNA and miRNA sequencing was extracted with the RNAqueous® Total RNA Isolation Kit (Cat. #AM1912; Ambion, Austin, TX, US) and the mirVana™ miRNA Isolation Kit (Cat. #AM1560; Ambion), respectively, according to the corresponding manufacturer’s protocol. The integrity of the total RNA was assessed using the Agilent BioAnalyzer 2100 (Agilent Technologies, Santa Clara, CA, USA).

### Library preparation and RNA sequencing

A total RNA pool of equal molar ratios was established from each group. The pooled RNA samples were purified with an RNeasy Micro Kit (Cat. #74004; QIAGEN, Venlo, Netherlands) for cDNA library preparation (approximately 3 μg of total RNA). Poly(A) mRNA isolation; first- and second-strand cDNA syntheses; and fragment, connecting adapter, and cDNA library preparation were performed sequentially with the TruSeq RNA Sample Prep Kit (Cat. #RS-122-2002; Illumina, San Diego, CA, USA) following the manufacturer’s protocol, and then each sample product was loaded onto flow cell channels of the Illumina High-Seq 2000 platform for paired-end 100-bp sequencing. The average insert size for the paired-end libraries was 400 bp (from 350 to 450 bp).

### Mapping and alignment of sequence reads

The CLC Genomics Workbench 4.8 (QIAGEN) was used to arrange the raw reads of the RNA-Seq. After the low-quality reads had been trimmed and the adapters had been removed, the clean reads were aligned onto the whole reference genome using TopHat[26], and two mismatches were allowed for the 100-bp reads in each alignment.

### Differential gene analysis of the RNA-Seq

The number of fragments per kilobase of exon length million mapped reads (FPKM) analyzed by Cufflink (version 2.0.2) was used as the value of the normalized gene expression. Gene expression differences were evaluated using the Fisher exact test after the total number of mapped reads in each lane had been normalized using the upper-quartile normalization method. Differentially expressed genes (DEGs) were identified using the statistical significance of the absolute value of fold change ≥2.0 and *P*≤ 0.05 between the Chinese pig group and the introduced pig group.

### Gene Ontology and Kyoto Encyclopedia of Genes and Genomes annotation

The DEGs were classified for the categories using the annotation of GO and KEGG pathways with the DAVID online software (http://david.abcc.ncifcrf.gov/home.jsp); to accomplish this, the official gene symbol for each DEG was uploaded, and the human with the maximum number of annotations in the David database was used. GO terms (BP (biological process), CC (cellular component), and MF (molecular function)) and KEGG pathways with a *P*-adjusted (Benjamini) less than 0.05 were considered to be significantly enriched with DEGs.

### Validation of DEGs by quantitative real-time PCR

The DEGs identified by the above-described method were validated using quantitative real-time PCR (qPCR). Primers designed for the qPCR spanned the exon-exon boundaries. *GAPDH* was used as a reference control. The information on the primers is listed in Table 1. Quantitative real-time PCR was performed using SuperReal PreMix Plus (SYBR Green) (FP205, TIANGEN BIOTECH, Beijing, China) on the CFX96 Real-Time System (BIO-RAD, Hercules, CA, USA). The quantitative real-time PCR program started with a 15min denaturation step at 95°C, followed by 40 cycles of 10s of denaturation at 95°C, 20s of annealing at 50–60°C and 20s of elongation at 72°C. Gene expression levels were calculated as previously described[27].

**Table 1:** Primers used in qPCR for eight genes.

GenBank accession number	Gene name	Sequence of primer 5′→3′	Product size (bp)	TM (°C)
NM_001244489.1	IRS1	F:AGTTTCCAGAAGCAGCCAGA	147	58
		R:ACCATCTACTGAGGAGGAAG		
LOC100512885	CARNS1	F:CAGCAAGAAGTTCGTGTGGGAG	138	60
		R:TCATCCCTCTGGTGCTCCGTTA		
HQ403607	ANKRD2	F: AGACACCTGCGACCAGTTC	175	60
		R: GAGTTTCACCACCTCCAAGT		
NM_001123091	CAV2	F: TCTCTTTGCCACACTCAGC	110	60
		R: CGTCTGTCACACTCTTCCA		
XM_011748904	MYOZ2	F: CCCTAAACTTTTCAAGCCTG	159	60
		R: ATGAACCTGGGATCTGTGAG		
NM_001170517	ACTC1	F: CAGGTCATCACTATTGGCA	156	60
		R: ATTGTTAGCATACAGGTCC		
XM_001924661	SORBS1	F:TGGTGATACGCAAGTGGAA	130	56
		R:ATAGGTGATGGGGAAGATG		
NM_214200.2	PLIN2	F:CATTGCCAACACTTACGC	136	60
		R:AGTAGTCGTCATAGCATCTT		
AF017079	GAPDH	F:GGTCACCAGGGCTGCTTTTA	134	56–63
		R:CCTTGACTGTGCCGTGGAAC		

### Library preparation and sequencing of miRNAs

The miRNA libraries were constructed according to the TruSeq Small RNA Sample Preparation protocol (Part #15004197 Rev. A, Illumina). The Illumina special RNA adapters were ligated to the small RNA molecules by the T4 RNA Ligase 2 (New England BioLabs, Ipswich, MA, USA). The small RNA ligated with the adapters was subsequently transcribed into the cDNA with SuperScript II Reverse Transcriptase (Cat. #18064014, Invitrogen, Grand Island, NY, USA), and then, PCR amplification of 11 cycles was performed with special primers (RP1 and RPIX, Illumina) corresponding to the ends of the adapters. After purification with a gel and validation byQubit^®^ 2.0 Fluorometer (Invitrogen) and Agilent BioAnalyzer 2100 (Agilent Technologies), the amplified cDNA constructs were sequenced according to the Illumina High-Seq 2000 platform with single-end 50bp sequencing protocols. The library sizes ranged from 140 bp to 160 bp, and four cDNA libraries were constructed, one for each pooled sample from the six pigs of each breed, with the same samples as used for the previously described RNA-seq.

### Analysis of miRNA-Seq data

The raw sequence reads were obtained with an Illumina Genome Analyzer at SBC-Shanghai, China. The Fastx (FastX_Toolkit v. 0.0.13.2) was applied to obtain clean reads from the raw data by removing the joint sequences, low-quality fragments, and sequences of <18 nucleotides (nt) in length. The CLC GenomicsWorkbench 5.5 software was used to align the sequences to the Sanger miRBase v19.0, and during this process, no mismatch was allowed. In addition, other noncoding RNA databases such as ncRNA, piRNA, and Rfam databases were used for alignment, and two base mismatches or the shortening or extension of both ends by two bases was permitted in the target sequences. The clean sequence reads were aligned with miRBase v19.

Annotated reads were classified according to their sources, and the known miRNAs were identified according to the various types of RNA molecules. DEGseq R Package with Perl script was used to perform comparative analysis of the expression levels of two samples to obtain the differentially expressed (DE) miRNAs.

To identify potential novel miRNA, we further analyzed the small RNA tags that could not be matched to the known miRNAs. The miRCat tool in the sRNA Toolkit software package was applied to predict novel miRNAs. The hairpin structure, as a marker of miRNA precursor, could be used to predict novel miRNA by analyzing the secondary structure.

miRanda version 3.1 (http://www.microrna.org/microrna/getMirnaForm) and Targetscan 5.1 (http://www.targetscan.org/) was applied to target gene prediction.

### MiRNA validation via stem-loop qPCR

Stem-loop qPCR, which has been described elsewhere[28], was used to validate the conserved and novel miRNAs. Briefly, the assay was performed using stem-loop reverse-transcription (RT)-PCR followed by SYBR Green Real-time PCR analysis. For the RT-PCR, we applied the miRcute miRNA First-Strand cDNA Synthesis Kit (KR201, TIANGEN BIOTECH, Beijing, China), which was used according to the manufacturer’s instructions. Porcine U6 snRNA was used as an internal control, and all reactions were run in triplicate. Gene expression levels were calculated using the 2^-△△Ct^ method as previously described. The primer sequences of the selected miRNAs are listed in Table 2.

**Table 2:** The qPCR primers of seven microRNAs (miRNAs).

miRNA name	miRNA primer sequence (5′→3′)
miR-4332	AAAAACACGGCCGCCGCCGG
miR-451	AACCGTTACCATTACTGAG
miR-497	CAGCAGCACACTGTGGTTTG
miR-196a	GGCGGGTAGGTAGTTTCA
miR-499	GGAAGACTTGCAGTGATGTT
miR-29C	GCACCATTTGAAATCGGTTA
miR29b	TAGCACCATTTGAAATCAGTGTT
U6	GCTTCGGCAGCACATATACT
miRNA universal reverse primer	GCGAGCACAGAATTAATACGAC

### Combined analysis of DEGs and DE miRNAs

We searched for DEGs from the target genes of the DE miRNAs and identified those genes with differential expression and those that were targets of the miRNA. Cytoscape mapping software was used to draft the network of the miRNAs and the DEGs.

## Results

### Body weight and IMF content

The results of the body weight and IMF content for the four pig breeds (DSP, TP, LL, and YY) are listed in Table 3. The DSP and the TP had significantly lower body weights and higher IMF than the LL and the YY (P < 0.01). The results indicated that the muscle tissues were right for searching genes related to muscle growth and lipid deposition.

**Table 3:** Intramuscular fat (IMF) content of the *longissimus dorsi* muscle in four pig breeds (mean ± SE).

Pig breed	DSP	TP	LL	YY
Number of samples	6	6	6	6
Weight of pigs	74.50 ± 1.05 ^a^	30.53 ± 0.98 ^c^	103.6 ± 1.68 ^e^	105.32 ± 1.89 ^e^
IMF content	2.98 ± 0.17 ^a^	2.78 ± 0.10 ^a^	1.26 ± 0.11 ^c^	1.10 ± 0.09 ^c^

Note: Different superscript letters (a, c, and e) within a row denote significant differences between groups; the same letter denotes no difference. DSP, Diannan Small-ear pig; TP, Tibetan pig; LL, Landrace; YY, Yorkshire.

### Transcriptome of LDmuscle via RNA-seq

**Fig 1:**
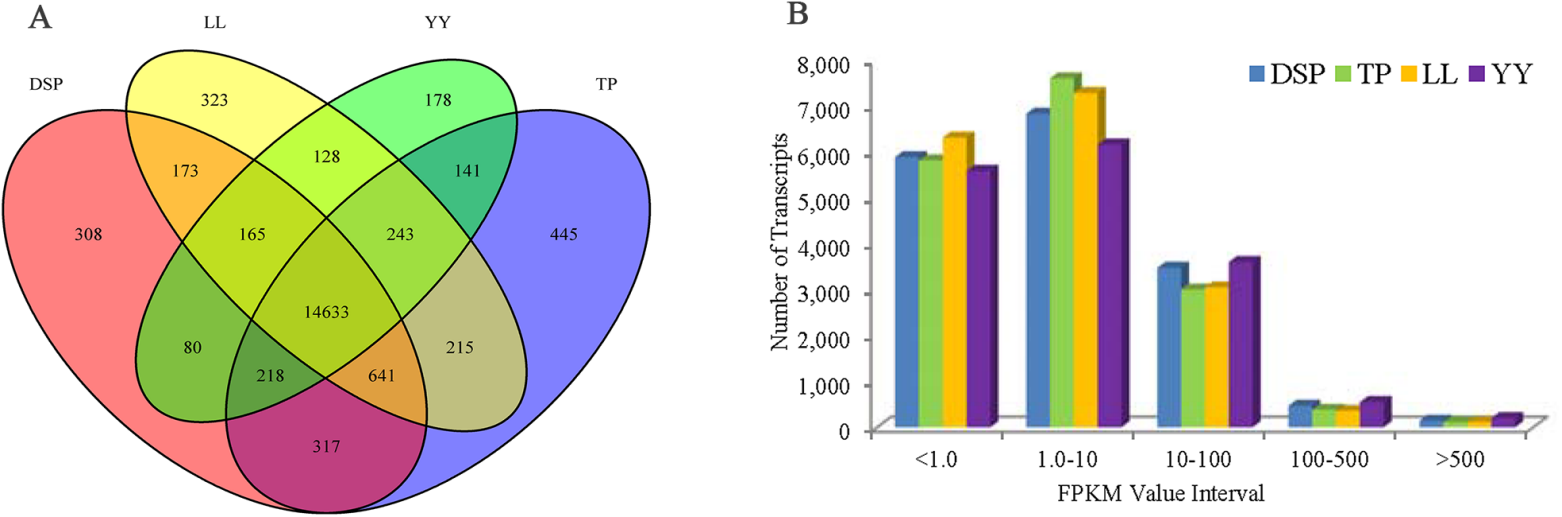
Summary of RNA sequence (RNA-Seq) mapping data. A: Venn diagrams of the number of genes expressed in each sample. B: The number of detected genes with different expression levels against the range of fragments per kilobase of exon length million mapped reads (FPKM) values.

In this study, 62.83 M to 77.17 M raw reads were generated for each sample, 79.31% to 82.21% of the clean reads were aligned with the pig reference genome (*Sus scrofa* 10.2) (Table 4), and 56.73% to 62.76% of the clean reads were distributed in coding regions (S1 Fig). The expression values (FPKM) of all calculated genes ranged from 2.43E-9 to 413,678 and had a median of 4.38 to 6.68 for each sample (S2 Fig). A total of 18,208 genes were detected as expressed in the pig LD muscle tissues, of which, 14,633 genes exhibited shared expression in the four samples (Fig 1A).

**Table 4:** Summary of sequencing reads aligned with the *Sus scrofa* genome and annotated genes.

sample	DSP	TP	LL	YY
Total reads	67,551,674	64,192,798	62,832,228	77,170,732
Total base pairs	6,755,167,400	6,419,279,800	6,283,222,800	7,717,073,200
Adaptor trimmed reads	64,320,230(95.22%)	54,030,697(84.17%)	59,947,706(95.41%)	64,665,945(83.80%)
Total clean reads	61,689,908(91.32%)	52,348,492(81.55%)	57,576,024(91.63%)	62,553,794(81.06%)
Total mapped reads	49,578,516(80.37%)	41,933,461(80.10%)	47,335,786(82.21%)	49,609,260(79.31%)
Unique matched reads	45,429,590(73.64%)	38,866,373(74.25%)	43,213,366(75.05%)	46,127,948(73.41%)
Multimatched reads	4,149,316(6.73%)	3,068,287(5.86%)	4,122,651 (7.16%)	3,483,050(5.57%)
Total unmapped reads	12,111,392(17.93%)	10,415,031 (16.22%)	10,240,238 (16.30%)	12,944,534(16.77%)

DSP, Diannan Small-ear pig; TP, Tibetan pig; LL, Landrace; YY, Yorkshire.

The detected genes were distributed in all chromosomal regions, and the genome coverage was plotted along the chromosome based on the expression of genes (S3 Fig). If the FPKM values of all the detected genes were divided into five intervals, less than 1.0, 1 to 10, 10 to 100, 100 to 500, and more than 500, the distribution of the FPKM of the genes was shown to be similar among the four samples (Fig 1B).

### DEGs and analysis

**Fig 2:**
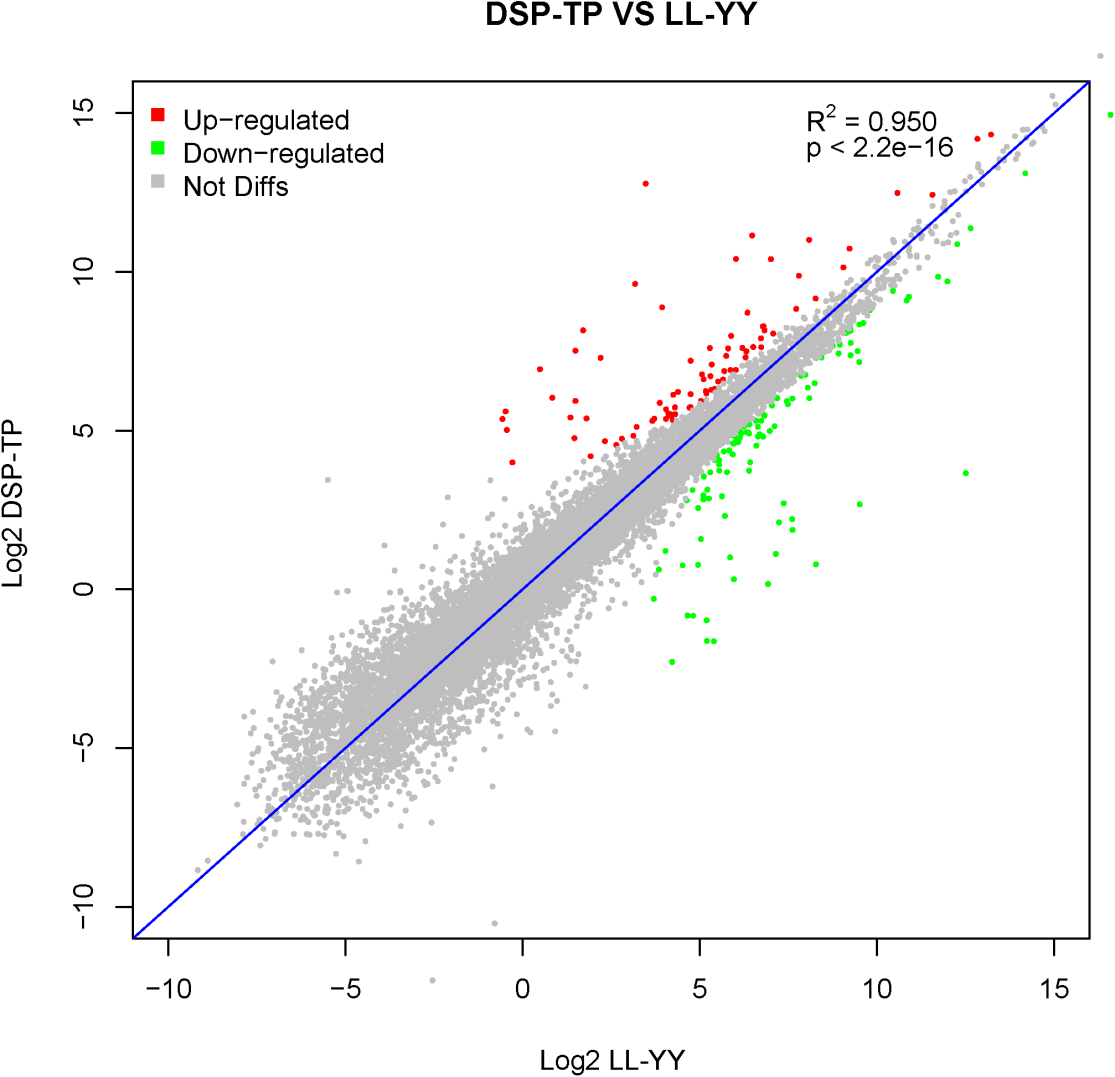
Comparison of the transcript expression levels between the Diannan Small-ear pig-Tibetan pig (DSP-TP) and Landrace-Yorkshire pig (LL-YY) groups. The vertical axis represents the Log2 fragments per kilobase of exon length million mapped reads (FPKM) in the DSP-TP group, and the horizontal axis represents the Log2 FPKM in the LL-YY group. The differentially expressed genes (DEGs) were filtered using P ≤ 0.05 and |log2ratio| ≥ 1 as a threshold. The red points represent upregulated genes, and the green points indicate downregulated genes. The gray spots represent no significant difference between the DSP-TP and LL-YY groups.

**Fig 3:**
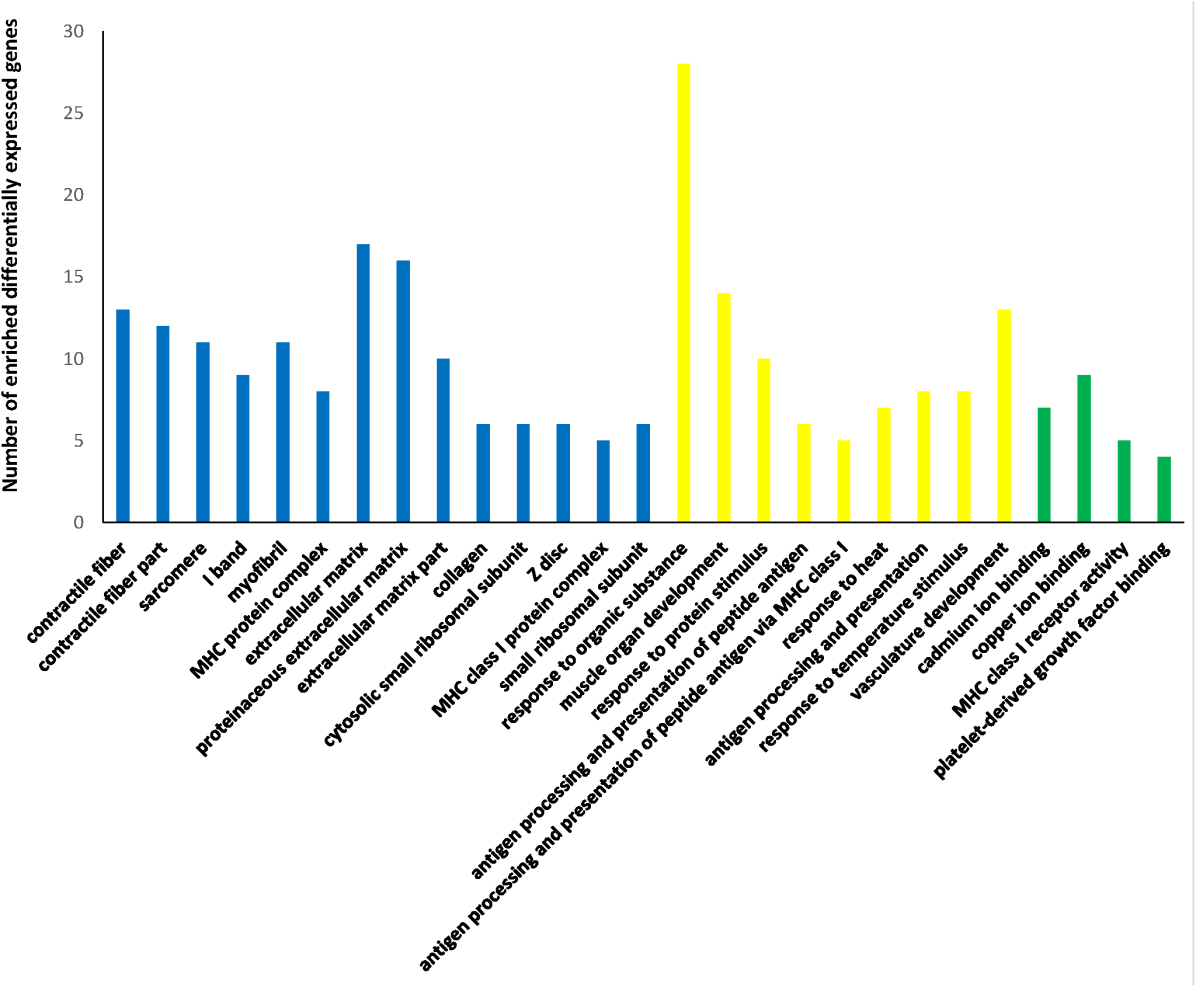
The significantly enriched Gene Ontology (GO) terms of differentially expressed genes. The blue clusters represent the cellular component, the yellow clusters represent the biological process, and the green clusters represent the molecular function of the GO terms.

The DSP and the TP were treated as the Chinese pig group (DSP-TP), which was characterized by slower growth and more lipid deposition than the LL and YY, which served as the introduced pig group (LL-YY). When the filter criteria of fold changes ≥ 2 and P ≤ 0.05 was applied, 315 DEGs were acquired from the DSP-TP and the LL-YY combined. Of these DEGs, 140 showed upregulated and 175 downregulated expression in the DSP-TP group (Fig 2 and S1 Table). Of the 315 DEGs, 240 were annotated in the David database and classified according to 178 GO terms. According to the criterion for the number of enriched DEGs (count ≥ 2 and *P*-adjusted (Benjamini) ≤ 0.05), 27 GO terms were obtained, and the terms were mainly associated with muscle fiber contraction, cadmium ion binding, response to organic substance, contractile fiber part, muscle organ development, and regulation of the lipid metabolic process(Fig 3).

Based on functional annotation, 85 DEGs (S2 Table) were related to the muscle growth, which involved the GO terms of muscle organ development, growth factor binding, collagen fibril organization, actin cytoskeleton, myofibril, etc. In the 85 DEGs, three genes, ryanodine receptor 3 (*RYR3*), mannose receptor C type 2 (*MRC2*), and activating transcription factor 3 (*ATF3*), had prominent differential expression with fold changes ≥ 5 (or ≤ 0.20) and a false discovery rate (FDR) ≤ 0.001 between the DSP-TP and LL-YY groups (Table 5). *RYR3* plays an important role in muscle cellular calcium signaling and in triggering muscle contractions[29]. *MRC2* encodes a protein that is a member of the mannose receptor family and that plays a role in the phagocytosis of pathogens[30]and resolution of inflammation[31]. *ATF3* encodes cAMP-dependent transcription factor ATF-3, which is related to the positive regulation of cell proliferation and skeletal muscle cell differentiation[32]. Based on functional analysis, 27 DEGs (S3 Table) were identified that are associated with lipid metabolism or deposition according to the GO terms of lipid metabolic process, fatty acid biosynthetic process, response to steroid hormone stimulus, etc. In the 27 DEGs, four genes, estrogen receptor 1 (*ESR1*), low density lipoprotein receptor (*LDLR*), stearoyl-CoA desaturase (*SCD*), and fatty acid synthase (*FASN*), exhibited prominent differential expression with fold changes ≥ 5 (or ≤ 0.20) and FDR ≤ 0.001 between the DSP-TP and LL-YY groups(Table 6). *ESR1* is essential for sexual development and reproductive functions and has an effect on some biological processes, such as in the leptin signaling pathway and in steroid binding. *LDLR* is involved in the bile secretion signaling pathway, which is essential for the digestion and absorption of fat, and is a cell-surface receptor related to the transport of plasma cholesterol into cells by endocytosis. The intracellular free cholesterol is used for cell proliferation and synthesis of steroidal hormone and bile acid salt[33]. *SCD* is involved in the biosynthesis of unsaturated fatty acids. The main function of the enzyme encoded by *FASN* is to catalyze the synthesis of palmitate from acetyl-CoA and malonyl-CoA, in the presence of NADPH, into long-chain saturated fatty acids. In addition, both *SCD* and *FASN* participate in the fatty acid metabolism pathway.

**Table 5:** Differentially expressed genes with fold change ≥5 (or≤0.20) and false discovery rate (FDR) ≤0.001 that are related to muscle growth.

Gene ID	Gene name	Chr.	DSP-TP FPKM	LL-YY FPKM	Fold change	FDR	UP/DOWN regulation
ENSSSCG00000002250	*RYR3*	7	521.98	15.32	34.08	5.9E-128	UP
ENSSSCG00000017300	*MRC2*	12	315.88	3.27	96.62	1.03E-84	UP
ENSSSCG00000015595	*ATF3*	9	214.37	39.03	5.49	1.5E-27	UP

DSP, Diannan Small-ear pig; FPKM, fragments per kilobase of exon length million mapped reads; TP, Tibetan pig; LL, Landrace; YY, Yorkshire.

**Table 6:** Differentially expressed genes with fold change ≥5 (or ≤0.20) and false discovery rate (FDR) ≤0.001 that are related to lipid deposition.

Gene ID	Gene name	Chr.	DSP-TP FPKM	LL-YY FPKM	Fold change	FDR	UP/DOWN regulation
ENSSSCG00000025777	*ESR1*	1	2505.61	89.25	28.07	0	UP
ENSSSCG00000028833	*LDLR*	GL896507.1	231.18	0.00	23,117,575	2.78E-65	UP
ENSSSCG00000010554	*SCD*	14	72.46	1.79	40.47	1.99E-17	UP
ENSSSCG00000029944	*FASN*	12	29.96	2.76	10.86	7.08E-05	UP

DSP, Diannan Small-ear pig; FPKM, fragments per kilobase of exon length million mapped reads; LL, Landrace; TP, Tibetan pig; YY, Yorkshire.

### The data of miRNA-seq

**Fig 4:**
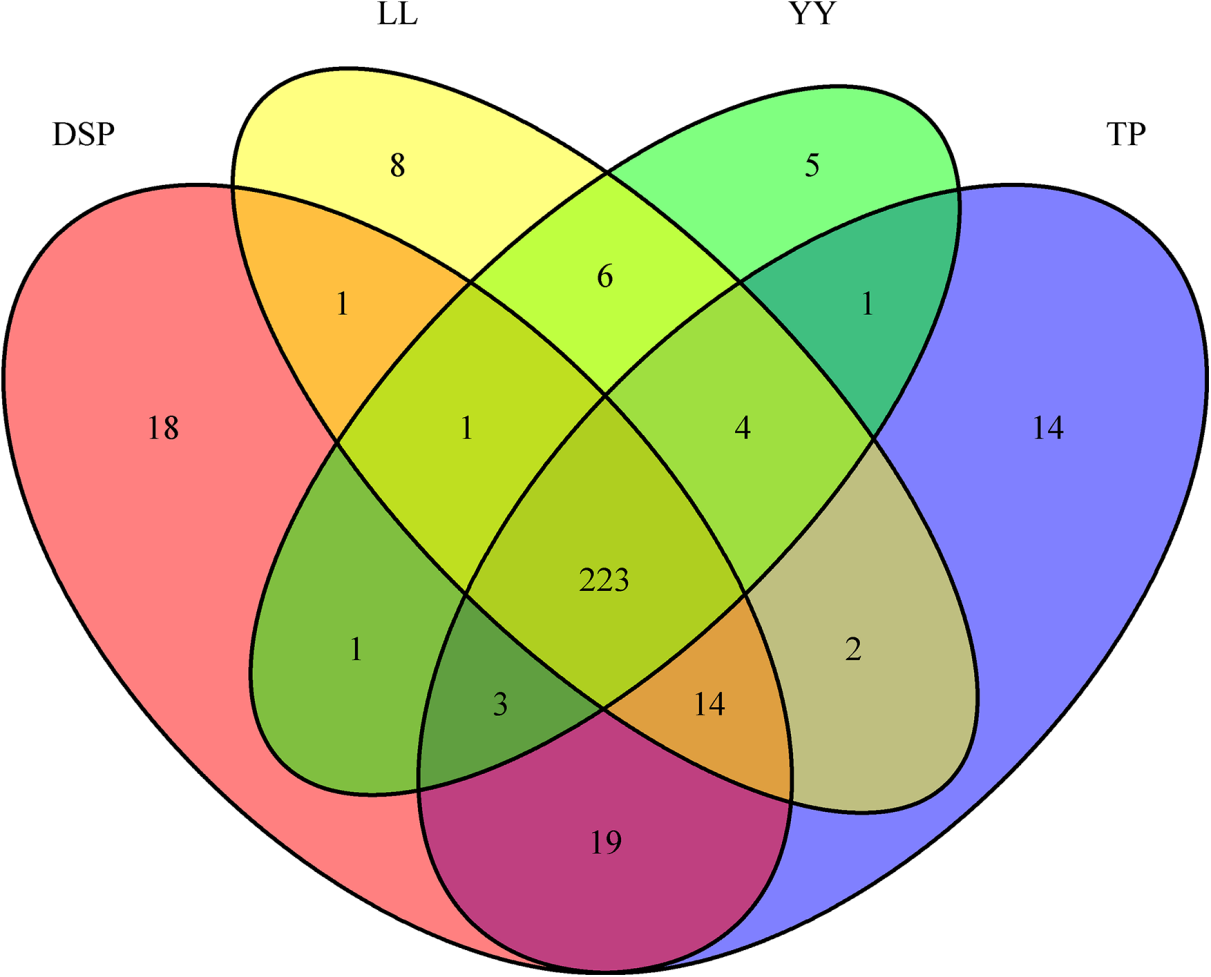
Venn diagram of expressed miRNA numbers in each sample.

We obtained 2.98 M to 7.09 M clean reads from each of the four small RNA libraries (S4 Table). The number of reads with 20- to 23-nt sequences was significantly greater than those reads of shorter or longer sequences. More than half of the sequences were 22 nt in length, which coincided with the known specificity of Dicer processing and the features of mature miRNAs. By aligning the sequences with different databases, the clean reads were distributed in the miRBase (S. scrofa) (approximately73.17%–90.11%), ncRNA (approximately 0.41%–1.96%), piRNA (approximately 0.13%–1.25%), and Rfam (approximately 5.48%–20.92%) databases (S5 Table). More than half of the annotation reads were acquired from miRBase (*S*. *scrofa*). Through analysis, we obtained 320 positive miRNAs in the muscle tissues, including 271 known pig miRNAs (S6 Table) and 49 novel miRNAs (S7 Table), which were predicted because of the associated canonical hairpin structure (S4 Fig). The Venn diagrams of the expressed miRNA numbers showed that 223 miRNAs shared expression in the four samples (Fig 4).

### DE miRNAs between Chinese pig and introduced pig groups

**Fig 5:**
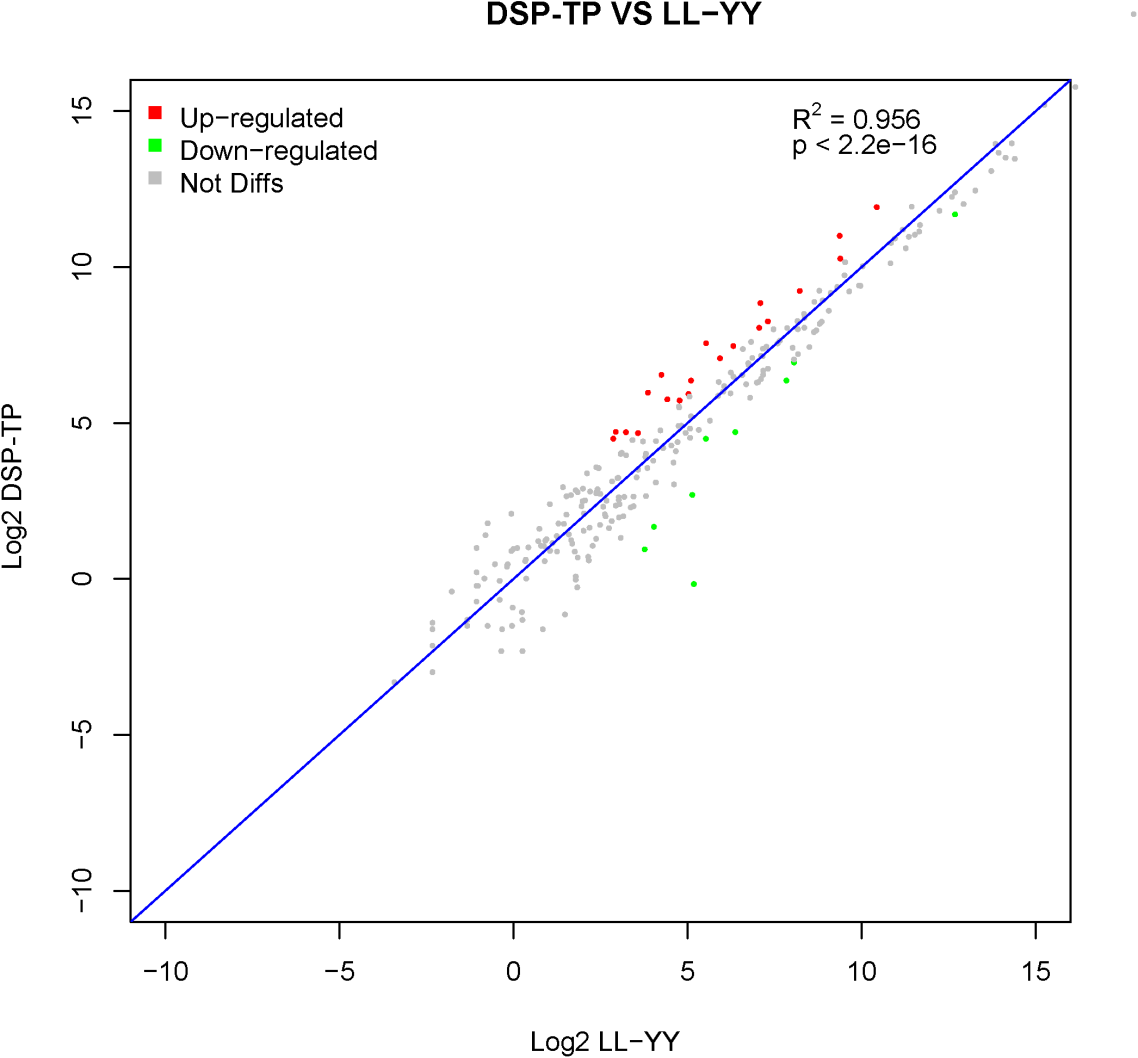
Comparison of transcript expression levels between Diannan Small-ear pig-Tibetan pig (DSP-TP) and Landrace-Yorkshire (LL-YY) groups. The vertical axis represents the Log2 fragments per kilobase of exon per million fragments mapped (FPKM) in the DSP-TP and the horizontal axis represents the Log2 FPKM in the LL-YY. Differentially expressed (DE) miRNAs were filtered using P ≤0.01 and |log2ratio| ≥1 as a threshold. The red points represent upregulated miRNAs, and the green points indicate downregulated miRNAs. The gray spots represent no significant differences between the DSP-TP and LL-YY samples.

Based on the criteria of fold changes ≥2 and P ≤0.01, 30 DE miRNAs were found between the Chinese pig group (DSP-TP) and the introduced pig group (LL-YY) in the 271 known miRNAs. Of these DE miRNAs, 20 were upregulated and 10 were downregulated in the DSP-TP group (Fig 5 and S8 Table).

### MiRNA target gene prediction and functional analysis

Using the TargetScan database, we predicted that the 20 upregulated and the 10 downregulated miRNAs had 1,893 (1,608 having gene names) (S9 Table) and 1,700 (1,412 having gene names) (S10 Table) target genes, respectively, according to the criterion of total energy ≤−25. To better understand the biological function, we performed KEGG functional annotations of the target genes using the DAVID database, and the results are shown in Tables 7 and 8. Those genes showing the highest counts are involved in pathways associated with cancer, for instance, the Wnt signaling pathway, which plays significant roles in the regulation of cell proliferation, embryonic axis specification, and morphogenetic movements[34]and was reported to inhibit the porcine adipogenic differentiation potential[35]. Notably, the targets of both the up- and downregulated miRNAs in the DSP-TP group are both involved in the mitogen-activated protein kinase 1 (MAPK) signaling pathway, which is closely related to the inhibition of lipogenesis[36]. Furthermore, the 14 putative target genes, regulated by the upregulated miRNAs, participate in the GnRH signaling pathway. Some target genes are also involved in the insulin-signaling pathway, which is related to proliferation and differentiation, fatty acid biosynthesis, and protein synthesis[37]. The enriched pathways showed that the DE miRNAs might be involved in the extracellular matrix (ECM)-receptor interaction[38]; glycine, serine, and threonine metabolism; the MAPK signaling pathway; pathways in cancer, such as the Wnt signaling pathway; focal adhesion; the calcium signaling; and other important biological processes. Some of the pathways were related to lipogenesis metabolism and adipocyte lineage commitment, as well as to cell development, growth, and proliferation.

**Table 7:** The Kyoto Encyclopedia of Genes and Genomes (KEGG) pathway enriched for targets of the 20 upregulated miRNAs in the Chinese pigs.

Signaling pathway term	Count	P value	False discovery rate
ECM-receptor interaction	19	2.79E-04	4.84E-02
Glycine, serine and threonine metabolism	10	1.01E-03	8.57E-02
MAPK signaling pathway	39	1.74E-03	9.80E-02
Pathways in cancer	44	4.37E-03	1.77E-01
Focal adhesion	30	4.84E-03	1.59E-01
Toll-like receptor signaling pathway	18	6.37E-03	1.73E-01
Chronic myeloid leukemia	14	1.27E-02	2.77E-01
Notch signaling pathway	10	1.93E-02	3.52E-01
Cell adhesion molecules (CAMs)	19	3.92E-02	5.46E-01
RIG-I-like receptor signaling pathway	12	4.41E-02	5.52E-01
Prostate cancer	14	4.59E-02	5.33E-01
VEGF signaling pathway	12	6.16E-02	6.11E-01
Small cell lung cancer	13	6.19E-02	5.83E-01
Insulin signaling pathway	18	8.16E-02	6.61E-01
Endocytosis	23	8.30E-02	6.43E-01
GnRH signaling pathway	14	8.61E-02	6.33E-01
Pancreatic cancer	11	9.70E-02	6.56E-01
Inositol phosphate metabolism	9	9.74E-02	6.37E-01
Glioma	10	9.77E-02	6.18E-01

**Table 8:** Kyoto Encyclopedia of Genes and Genomes (KEGG) pathway enriched for targets of the 10 downregulated miRNAs in the Chinese pigs.

Signaling pathway term	Count	P value	False discovery rate
Calcium signaling pathway	25	1.67E-03	2.62E-01
Notch signaling pathway	10	5.63E-03	4.00E-01
ECM-receptor interaction	13	1.65E-02	6.34E-01
Gap junction	12	5.40E-02	9.19E-01
MAPK signaling pathway	27	6.95E-02	9.26E-01
Alzheimer's disease	18	7.77E-02	9.13E-01
Arginine and proline metabolism	8	8.44E-02	8.98E-01
Focal adhesion	21	8.67E-02	8.71E-01
ABC transporters	7	9.40E-02	8.63E-01
Fructose and mannose metabolism	6	9.44E-02	8.34E-01
Arrhythmogenic right ventricular cardiomyopathy (ARVC)	10	9.48E-02	8.06E-01

### Validation of DEGs and DE miRNAs

**Fig 6:**
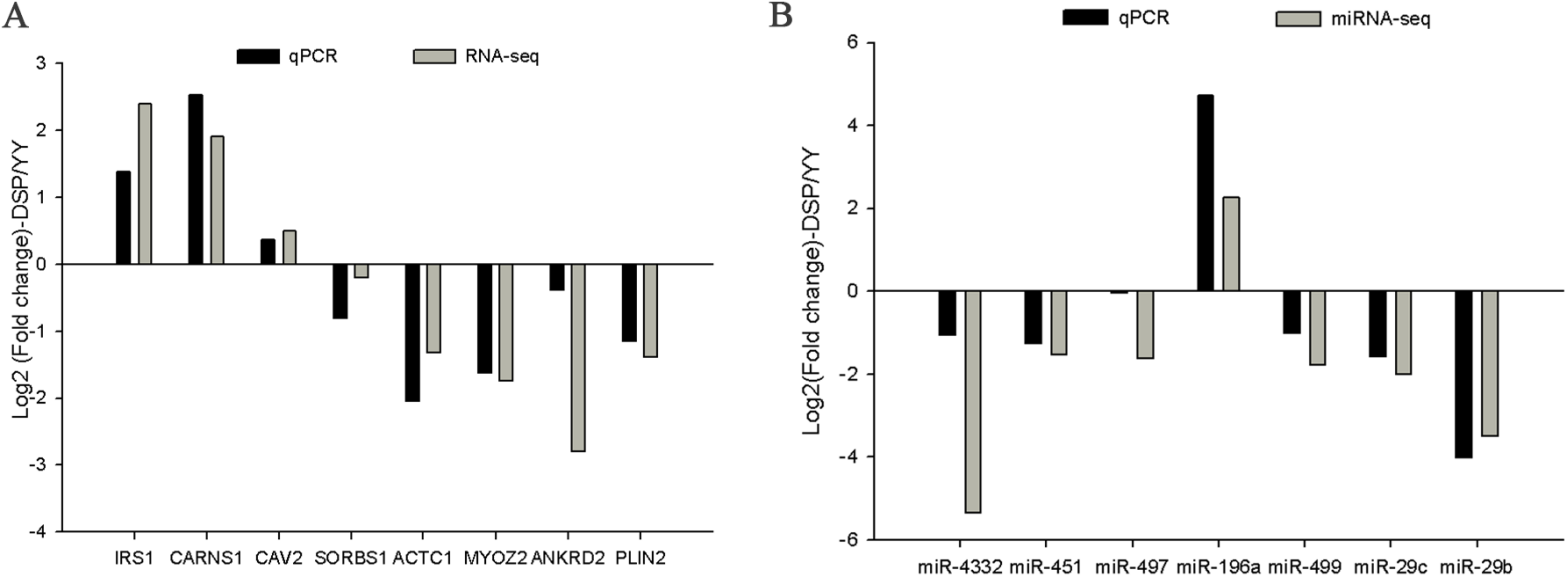
Eight differentially expressed genes (DEGs) (A) and seven differentially expressed (DE)microRNAs (miRNAs) (B) were validated by qPCR in *longissimus dorsi*(LD)muscle tissues.

We selected eight DEGs (*IRS1*, *CARNS1*, *CAV2*, *SORBS1*, *ACTC1*, *MYOZ2*, *ANKRD2*, and *PLIN2*) and seven DE miRNAs (miR-4332, miR-451, miR-497, miR-196a, miR-499, miR-29c, and miR-29b) randomly to validate the accuracy of the RNA-seq and miRNA-seq using qPCR, including the up- and downregulated genes and miRNAs. The expressions of five genes (*IRS1*, *CARNS1*, *MYOZ2*, *ANKRD2*, and *PLIN2*) and four miRNAs (miR-4332, miR-451, miR-196a, and miR-29b) were significantly different between the DSP and the YY (S5 and S6 Figs). Furthermore, the fold changes of the eight genes and the seven miRNAs in the qPCR and in the RNA-seq or miRNA-seq showed the same trends (Fig 6). The results indicated that the DEGs and DE miRNAs identified with NGS were reliable and efficient.

### Combined analysis of DEGs and DE miRNAs

When we combined the DE miRNAs and the DEGs, we found that 46 DEGs related to growth traits had a targeted relationship with 18 DE miRNAs, and 15 DEGs related to lipid deposition had a targeted relationship with 16 DE miRNAs. We drafted gene and miRNA regulatory networks to elaborate the molecular mechanisms of muscle growth (S7 Fig) and lipid deposition in pigs (S8 Fig). *CAV2* expression was upregulated in DSP-TP; this gene was the target of miR-29b and miR-122, which hadapproximately seven- and fivefold lower expression in the DSP-TP than in the LL-YY, respectively. The protein encoded by *CAV2* is involved in essential cellular functions, including signaling transduction, lipid metabolism, cellular growth control, and apoptosis. *MYOZ2* responsible for inhibiting calcineurin activity and then regulating the differentiation of muscle fibers[39] was a target of the miR-145-5p, which showed upregulated expression in the DSP-TP; therefore, the expression of the target gene was downregulated in the DSP-TP (P = 1.62E-62). As the targeted gene of miR-let-7c, *FRZB* participated in the Wnt signaling pathway and was related to cell development, growth, and proliferation. Our previous study showed that the expression of *FRZB* has a negative association with muscle growth and a positive association with fat deposition; therefore, *FRZB* is a major candidate gene for growth traits in pigs[40]. Therefore, we determined that the genes *CAV2*, *MYOZ2*, and *FRZB* and the miRNAs miR-29b, miR-122, miR-145-5p, and miR-let-7c are the key candidates for regulating muscle growth.

In the regulatory networks of lipid deposition, several important lipogenic DEGs, *FASN*, *SCD*, and *ESR1* were regulated by miR-4332, miR-182, miR-92b-3p, and miR-29c, respectively. The four miRNAs showed lower expression in the DSP-TP group, which suggests that these miRNAs have effects on lipid deposition by regulating their target genes. *ADORA1* (Adenosine receptor A1), which showed downregulated expression in the DSP-TP, was the target of three miRNAs, let-7a, let-7c, and let-7e.

## Discussion

In this study, the LD muscle samples from four pig breeds, comprising two Chinese breeds (DSP and TP) and two introduced breeds (LL and YY), were used tocompare the differences of the transcriptomes and the miRNAomics profiles. We found 315 DEGs that mainly enriched in GO terms associated with muscle fiber contraction, cadmium ion binding, contractile fiber part, muscle organ development, and regulation of the lipid metabolic process, etc. In previous studies, various numbers of DEGs (from 39 to 4331) were reported in pig muscle tissues with transcriptome analysis[3–8, 41, 42]. In addition of the technology itself, sample designs might contribute to the obvious variation in results. In present study, two mini-type pig breeds (DSP and TP) that both had special characteristics of high lipid deposition and slow body growth were selected to contrast with two lean-type pig breeds (LL and YY) that both had known traits of low lipid deposition and fast growth. Body weight and IMF content measured in this study (Table 3) confirmed the phenotype difference between the breeds. The key genes and miRNAs identified in this study had 6 overlap (*FABP3*, *HK2*, *PRKAG2*, *CEBPD*, miR-208b and miR-29c) with the DEGs and DE miRNAs reported by Jing, et al.[4] and 2 overlap (*STMN1* and *ID1*) with the DEGs reported by Zhao, et al.[5].

In the found 315 DEGs, the *H-FABP* (heart-type fatty acid-binding protein, or *FABP3*) showed the highest expression. An experiment on *H-FABP*-deficient mice found that *H-FABP* plays a crucial role in the uptake and oxidation of long-chain fatty acids (LCFAs)[43] and demonstrated that low expression of *H-FABP* resulted in lower LCFA oxidization. In this study, the expression of *H-FABP* in the LL-YY group was 2.13-fold higher than that in the DSP-TP group, which might have a negative effect on the IMF of LD muscle as described in pigs[44]and chickens[45].

In all, 85 DEGs, which accounted for about 25% of the total DEGs, were classified as being associated with muscle growth by GO terms analysis as follows: contractile fiber, muscle contraction, growth factor binding, transforming growth factor beta receptor signaling pathway, actin cytoskeleton organization, regulation of actin cytoskeleton skeletal system morphogenesis, etc. In the DEGs, the expression values of *RYR3*, *MRC2*, and *ATF-3* in the DSP-TP group were approximately 34-, 97- and 5.5-folds higher than in the LL-YY. Based on functional annotation, the genes of *RYR3*, *MRC2*, and *ATF-3* may be the main candidate genes that inhibit muscle growth in the DSP and TP.

We identified 27 DEG-related lipid deposition traits that accounted for about one-eleventh of the total number of DEGs. Of these, the genes of *LDLR*, *SCD*, and *FASN* dominated according to the value of fold-change and FDR. *LDLR* is a cell membrane glycoprotein that plays a critical role in cholesterol homeostasis and lipid metabolism in mammals[46–48]. In this study, the *LDLR*mRNA was only detected in the DSP-TP, which suggests that LDLR was necessary for maintaining the lipid homeostasis in the LD tissues. SCD is a rate-limiting enzyme involved in the biosynthesis of unsaturated fatty acids from saturated fatty acids[49], primarily the synthesis of oleic acid. The expression of SCD protein in LD muscle of hybrid populations (Large White × Landrace) has been positively associated with monounsaturated fatty acid content and total muscle fatty acids content[50]. *SCD* mRNA expression level in the LD muscle of the DSP-TP was approximately 40.5-fold higher than that of the LL-YY, which may be one reason for the higher IMF content in the DSP-TP compared to the LL-YY. Whether the monounsaturated fatty acids content is higher in the DSP-TP than in the LL-YY needs to be measured in further studies. *FASN* has a key role in catalyzing the formation of long-chain fatty acids. In this study, the expression of *FASN* was approximately 10-fold higher in the LD tissue of the DSP-TP than in the LL-YY group, which suggested stronger lipid deposition in Chinese local pigs. This is in agreement with the expression level of *FASN* in intramuscular preadipocytes from the Wujin pig (another local Chinese breed) and Landrace pigs, respectively[51]. In our data, the key lipogenic genes such as *FASN* and *SCD* had higher expression levels in the group with high-IMF content, which may be the reason for the stronger lipid deposition capacity in the Chinese pigs than the introduced pigs.

In microRNA transcriptome profiling, 30 DE miRNAs were obtained. Based on the RPKM values, the top four of the DE miRNAs were miR-let-7a, miR-99a, miR-374a-5p, and miR-148b-3p, whose sum of RPKM varied from approximately 2,035 to 9,865. Based on the fold change, the top five were miR-4332 (FC = 0.024), miR-29b (FC = 0.140), miR-92b-3p (FC = 0.183), miR-362 (FC = 5.406), and miR-122 (FC = 0.193). These nine miRNAs may be the most important miRNAs for regulating the genes for muscle growth and fat deposition in LD.

We analyzed the transcriptome profiling of differentially expressed genes and miRNAs selected from two groups for muscle growth and lipid metabolism. The 46 DEGs for muscle growth were involved in the targets of 18 DE miRNAs. These DEGs included crucial growth genes, such as *CAV2*, *MYOZ2*, and *FRZB*, etc. Previous studies have reported that *CAV2* downregulated cancer cell proliferation and could modulate cancer progression[52]. This may explain why *CAV2* was highly expressed in the DSP-TP, which had the slower growth ratio. *MYOZ22* has been reported to be a member of the muscle protein family that binds tocalcineurin[53]. In cardiac and skeletal muscle, the product of *MYOZ2* appears to influence the expression of calcineurin, which is required for the key processes of myocyte differentiation and conversion to the slow (oxidative) muscle phenotype and plays an important role in hypertrophic cardiomyopathy and skeletal muscle fiber differentiation[54]. Therefore, expression of *MYOZ2* can directly affect muscle growth. In our data, the expression of *MYOZ2* was higher in the LL-YY than in the DSP-TP, and this finding suggests that *MYOZ2* could stimulate muscle growth[55]. Furthermore, *FRZB* could antagonize the Wnt signaling pathway, which further inhibited muscle growth. The 15 DEGs related to lipid deposition belonged to the putative targets from 16 DE miRNAs. The genes *FASN*, *SCD*, and *ESR1*, which function to promote lipogenesis, may be regulated by miRNAs with downregulated expression in the DSP-TP, miR-4332, miR-182, miR-92b-3p, and miR-29c, which was coincident with the regulatory mechanism between gene expression and miRNA. *ADORA1* is known to play important roles in many metabolism processes, such as lipid catabolism[56],cell proliferation, and hormone secretion. *ADORA1* also had been confirmed to trigger lipolysis in rat[57] and human adipocytes[58] which suggests that its increased expression in the LL-YY is conducive to lipolysis. Five miRNAs, three highly expressed in the LL-YY (miR-let-7a, miR-let-7c, and miR-let-7e) and two highly expressed in the DSP-TP (miR-339 and miR-362), were identified as targeting *ADORA1* gene.

In conclusion, we obtained 315 DEGs in this study, and 85 and 27 of the DEGs were related to muscle growth and lipid metabolism, respectively. Some important muscle growth and lipid metabolism genes were identified. A total of 30 DE miRNAs and 49 novel miRNAs in porcine LD muscle were identified. By combining the miRNA- and the mRNA-Seq data, we were able to understand the regulatory relationship between DEGs and DE miRNAs. Some DE miRNAs, such as miR-29b, miR-122, miR-145-5p, let-7c, miR-4332, miR-182, miR-92b-3p, miR-29c,let-7a, and let-7e, and some DEGs such as *CAV2*, *MYOZ2*, *FRZB*,*FASN*, *SCD*, *ESR1*, and *ADORA1*, may be factors in the regulation of muscle growth and lipid deposition.

## Supporting Information

S1 Fig Distribution of clean reads in the pig genome. The different color represents different distribution region of clean reads.
(PDF)

S2 Fig Box-and-whisker plots of four samples. The box-and-whisker plots show log2 fragments per kilobase of exon length million mapped reads (FPKM) of each gene from the four sets of RNA-seq data. The black line in the box represents the median.
(PDF)

S3 Fig The fragments per kilobase of exon length million mapped reads (FPKM) value distribution of each gene in four samples from the chromosomes. The FPKM value distribution of each gene in four samples from chromosome 1 to 18 and chromosomes X and Y of the pig genome is shown in blue for each fatty and fast-growing sample and in red for each lean and slow-growing sample. Genome coverage of each sample was plotted in 10-kb windows along the chromosome. The blue and red peaks represent FPKM < 1 and FPKM > 1, respectively.
(PDF)

S4 Fig The structure of 49 predicted novel miRNAs. The green area in the structure represents the novel mature miRNA, and the purple area represents the miRNA*.
(PDF)

S5 Fig The expression of eight genes validated by qPCR in pig *longissimus dorsi*(LD) muscle tissue. The vertical axis represents the expression value of miRNA in pig LD and the horizontal axis represents names of eight genes. Error bars represent SE of expression. * *on the bars indicate significant differences (*P*<0.05) and *** indicate extremely significant differences (*P*<0.01) between DSP and YY breeds. DSP = Diannan Small-ear pig (n = 8). YY = Yorkshire (n = 8).
(PDF)

S6 Fig The expression of seven miRNAs validated by qPCR in pig *longissimus dorsi* (LD) muscle tissue. The vertical axis represents the expression value of miRNA in pig LD and the horizontal axis represents names of seven miRNAs. Error bars represent SE of expression. * *on the bars indicate significant differences (*P*<0.05) and *** indicate extremely significant differences (*P*<0.01) between DSP and YY breeds. DSP = Diannan Small-ear pig (n = 8). YY = Yorkshire (n = 8).
(PDF)

S7 Fig Network graphic of 18 differentially expressed miRNAs and 46 differentially expressed genes (DEGs) related to muscle growth. The pink circle represents the differential gene with the higher expression in the Diannan Small-ear pig-Tibetan pig (DSP-TP) group than that in the Landrace-Yorkshire (LL-YY) group, and the blue circle represents the differential gene with the lower expression. The red triangle represents differential miRNAs with higher expression in the DSP-TP group than that in the LL-YY group, and the green triangle represents the differential miRNAs with lower expression.
(PDF)

S8 Fig Network graphic of 16 differentiallyexpressed miRNAs and 15 differentially expressed genes (DEGs) related to lipid deposition. The pink circle represents the differential gene with the higher expression in the Diannan Small-ear pig-Tibetan pig (DSP-TP) group than that in the Landrace-Yorkshire (LL-YY) group, and the blue circle represents the differential gene with the lower expression. The red triangle represents the differential miRNAs with higher expression in the DSP-TP group than that in the LL-YY group, and the green triangle represents the differential miRNAs with lower expression.
(PDF)

S1 Table Differentially expressed genes between the Chinese pig group and the introduced pig group. (XLSX)

S2 Table The list of the 85 differentially expressed genes (DEGs) related to growth traits. (XLSX)

S3 Table The list of the 27 differentially expressed genes (DEGs) related to lipid deposition traits. (XLSX)

S4 Table The sequencing yield of the four libraries. (DOCX)

S5 Table The resource distribution of the reads. (XLSX)

S6 Table Identified known miRNAs between the Chinese pig and the introduced pig groups. (XLSX)

S7 Table Total novel miRNAs in the four pig libraries. (XLSX)

S8 Table The miRNAs that were differentially expressed between the Chinese and introduced pig groups. (XLSX)

S9 Table Predicted target genes of the upregulated miRNAs. (XLSX)

S10 Table Predicted target genes of the downregulated miRNAs. (XLSX)
