# Correction: Identification of Genes Related to Growth and Lipid Deposition from Transcriptome Profiles of Pig Muscle Tissue

**DOI:** 10.1371/journal.pone.0172930

**Published:** 2017-02-22

**Authors:** Zhixiu Wang, Qinggang Li, Yangzom Chamba, Bo Zhang, Peng Shang, Hao Zhang, Changxin Wu

The Data Availability statement for this paper is incorrect. The correct statement is: All relevant data are available within the paper and its Supporting Information files. RNA sequence data are available from the NCBI Gene Expression Omnibus (GEO) repository under the following accession numbers: GSE93023, GSE93021, and GSE93022.
