# Supplementary material for: Identification of Genes Related to Growth and Lipid Deposition from Transcriptome Profiles of Pig Muscle Tissue
Source: PLoS One. 2015 Oct 27;10(10):e0141138. doi: 10.1371/journal.pone.0141138 (PMC4624711; doi:10.1371/journal.pone.0141138)
Supplement: S1 Fig — The different color represents different distribution region of clean reads. (PDF) [file pone.0141138.s001.pdf]

S1 Fig. Distribution of clean reads in the pig genome

The different color represents different distribution region of clean reads.

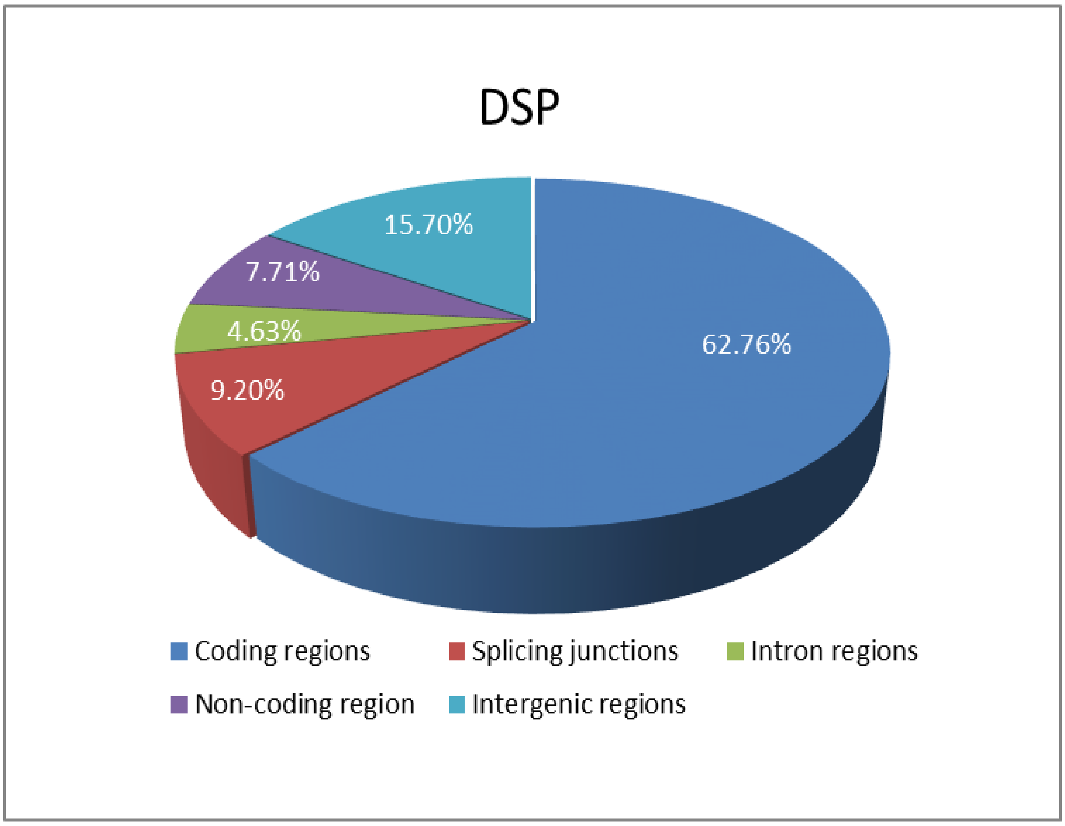

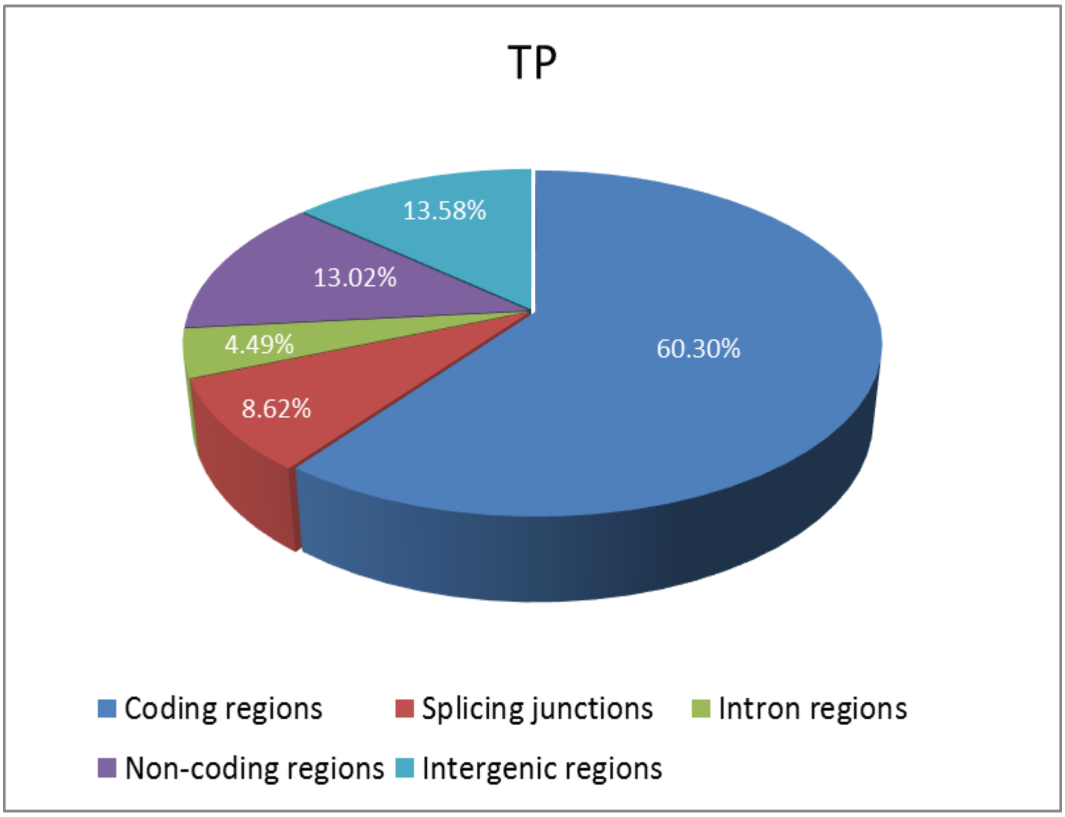

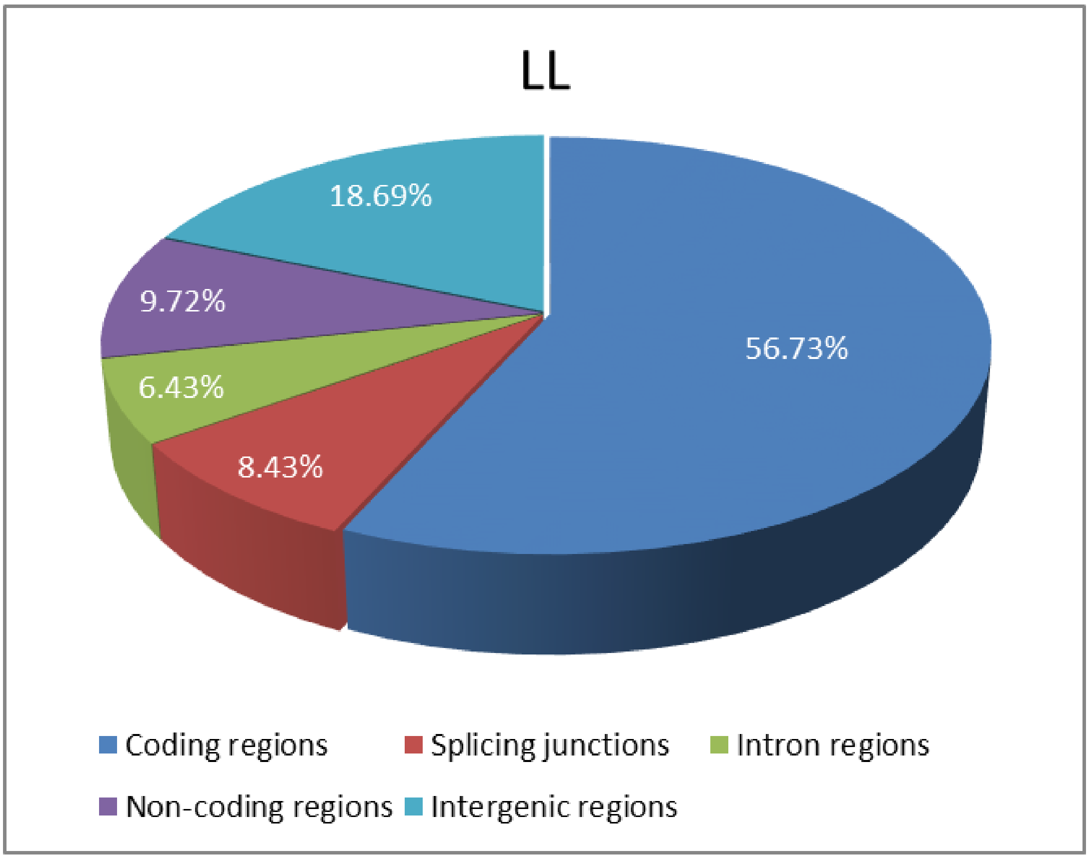
LL
18.69%
9.72%
6.43%
8.43%
56.73%
Coding regions
Splicing junctions
Intron regions
Non-coding regions
Intergenic regions

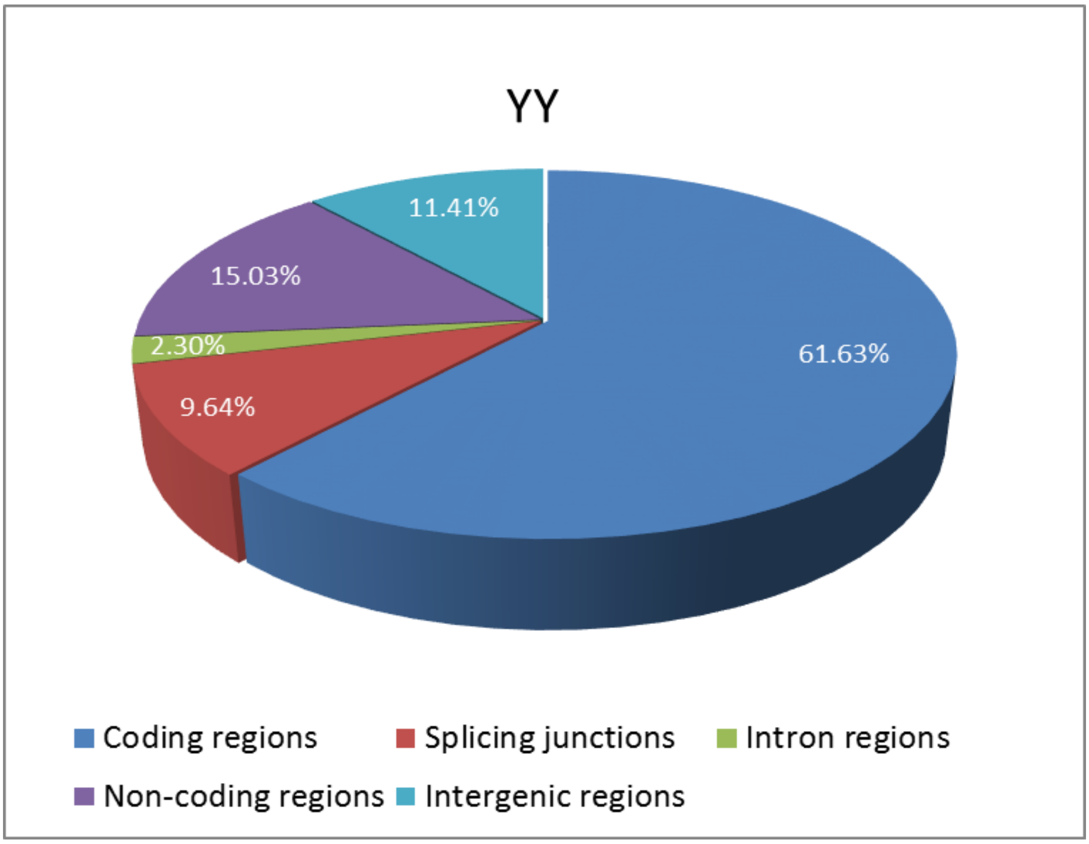
YY
11.41%
15.03%
2.30%
9.64%
61.63%
Coding regions
Splicing junctions
Intron regions
Non-coding regions
Intergenic regions
