# Supplementary material for: Identification of Genes Related to Growth and Lipid Deposition from Transcriptome Profiles of Pig Muscle Tissue
Source: PLoS One. 2015 Oct 27;10(10):e0141138. doi: 10.1371/journal.pone.0141138 (PMC4624711; doi:10.1371/journal.pone.0141138)
Supplement: S2 Fig — The box-and-whisker plots show log2 fragments per kilobase of exon length million mapped reads (FPKM) of each gene from the four sets of RNA-seq data. The black line in the box represents the median. (PDF) [file pone.0141138.s002.pdf]

S2 Fig. Box-and-whisker plots of four samples

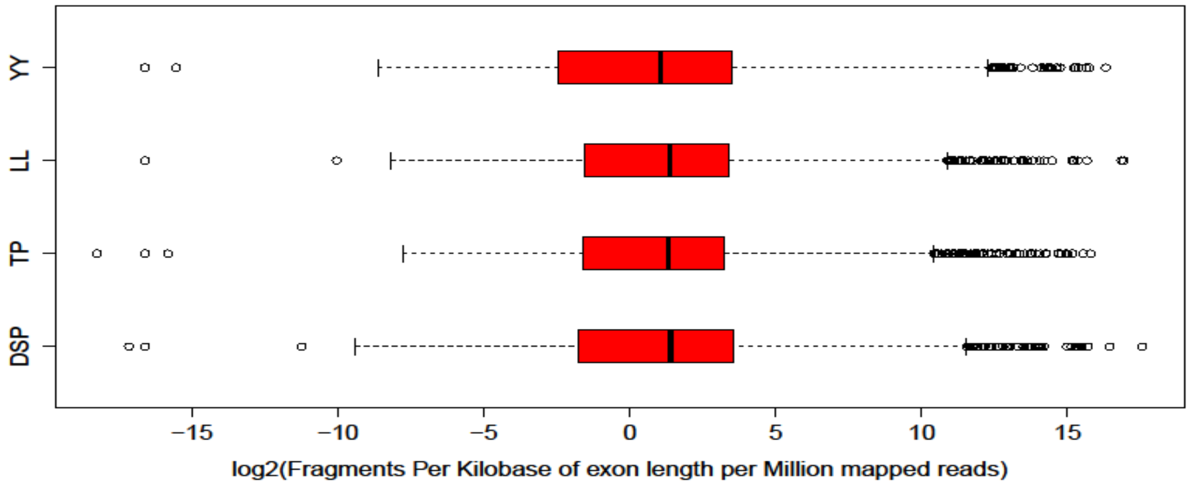

The box-and-whisker plots show log2 fragments per kilobase of exon length per million mapped reads (FPKM) of each gene from the four sets of RNA-seq data. The black line in the box represents the median.
