# Supplementary material for: Identification of Genes Related to Growth and Lipid Deposition from Transcriptome Profiles of Pig Muscle Tissue
Source: PLoS One. 2015 Oct 27;10(10):e0141138. doi: 10.1371/journal.pone.0141138 (PMC4624711; doi:10.1371/journal.pone.0141138)
Supplement: S3 Fig — The FPKM value distribution of each gene in four samples from chromosome 1 to 18 and chromosomes X and Y of the pig genome is shown in blue for each fatty and fast-growing sample and in red for each lean and slow-growing sample. Genome coverage of each sample was plotted in 10-kb windows along the chromosome. The blue and red peaks represent FPKM < 1 and FPKM > 1, respectively. (PDF) [file pone.0141138.s003.pdf]

S3 Fig. The fragments per kilobase of exon length million mapped reads (FPKM) value distribution of each gene in four samples from the chromosomes.

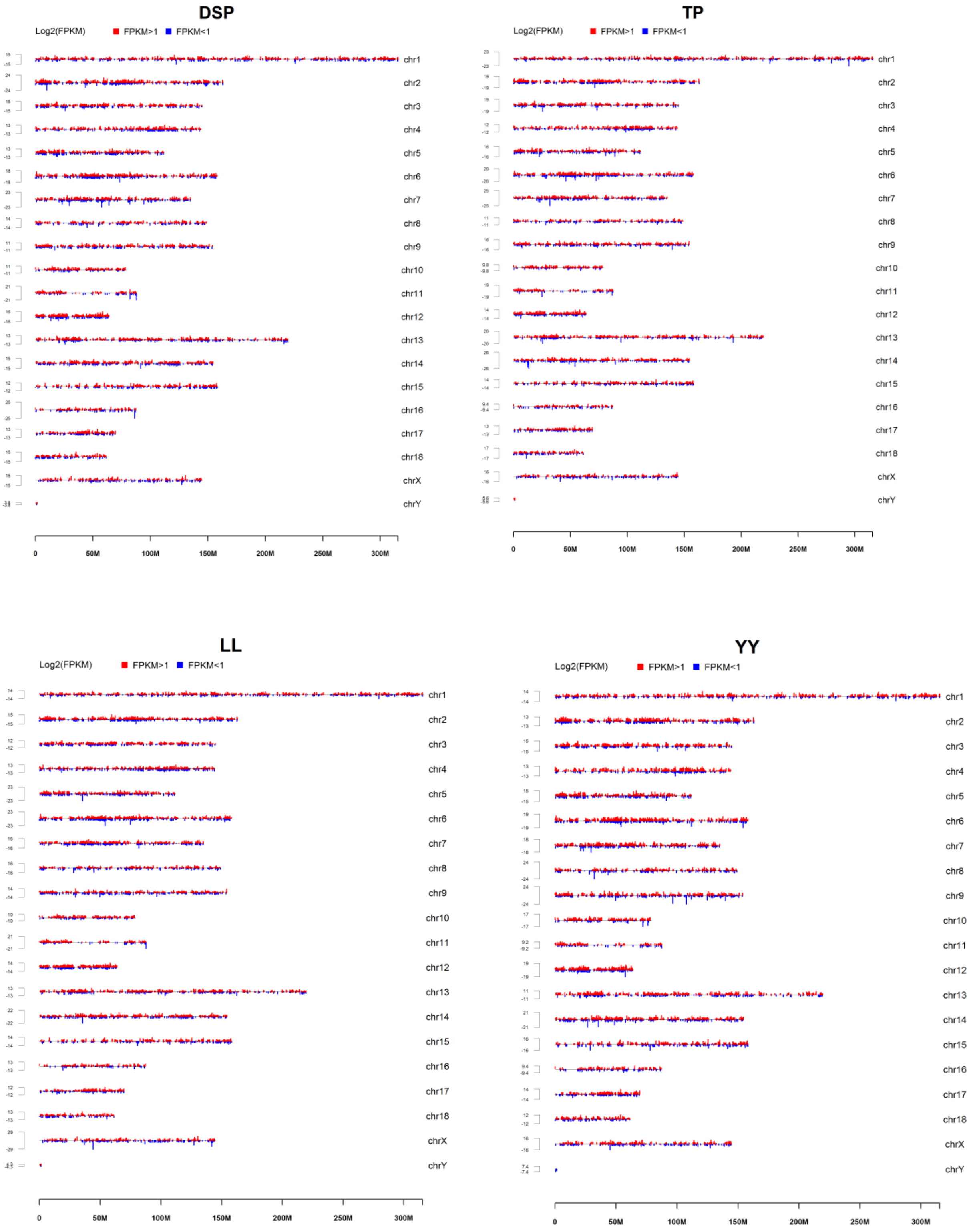

The FPKM value distribution of each gene in four samples from chromosome 1 to 18 and chromosomes X and Y of the pig genome is shown in blue for each fatty and fast-growing sample and

in red for each lean and slow-growing sample. Genome coverage of each sample was plotted in 10-kb windows along the chromosome. The blue and red peaks represent FPKM < 1 and FPKM > 1, respectively.
