# Supplementary material for: Identification of Genes Related to Growth and Lipid Deposition from Transcriptome Profiles of Pig Muscle Tissue
Source: PLoS One. 2015 Oct 27;10(10):e0141138. doi: 10.1371/journal.pone.0141138 (PMC4624711; doi:10.1371/journal.pone.0141138)
Supplement: S4 Fig — The green area in the structure represents the novel mature miRNA, and the purple area represents the miRNA*. (PDF) [file pone.0141138.s004.pdf]

S4.The structure of 49 predicted novel miRNAs

The green area in the structure represents the novel mature miRNA, and the purple area represents the miRNA*.

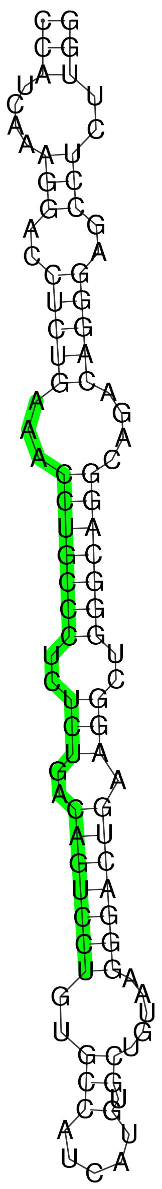

AAACCTGCCCTCTCTGACAGTCCT_chr1_154994061-154994084

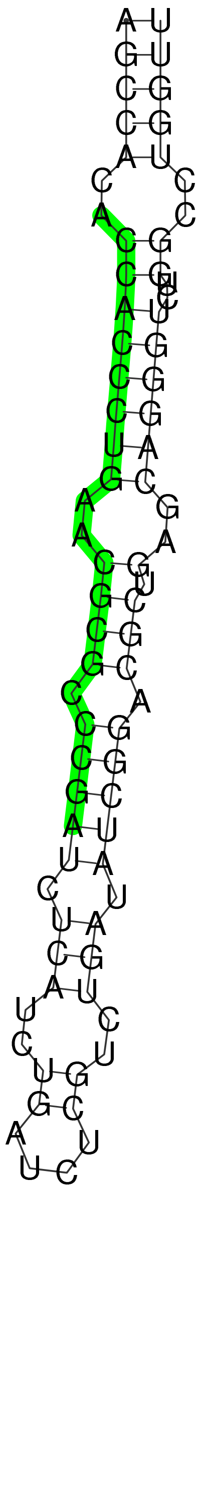

ACCACCCTGAACGCGCCCGA_chr13_139351583-139351602

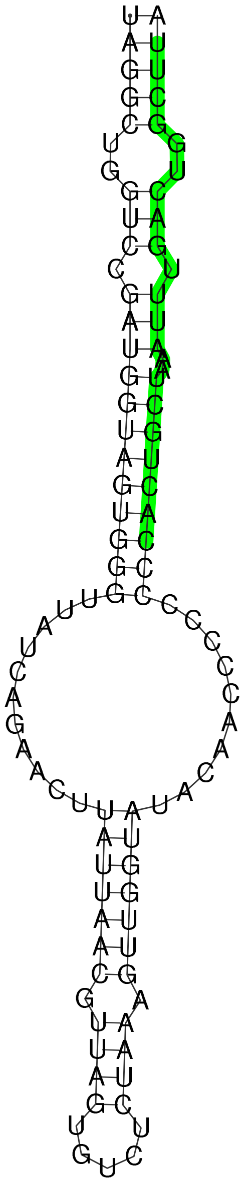

CACTGCTAAATTTGACTGGCTT_chr10_6017533-6017554

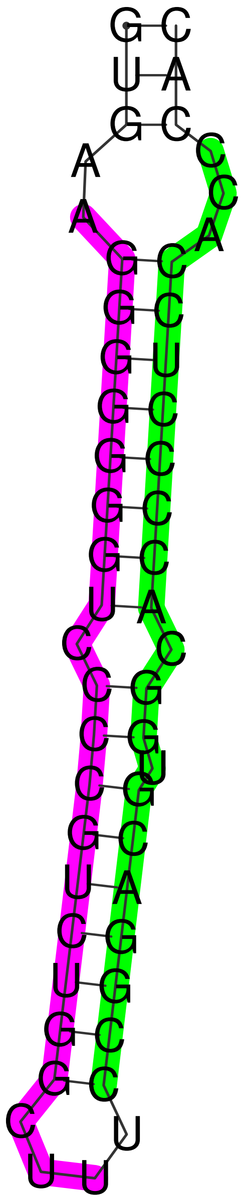

CCGGACGTGGCACCCCTCCACC_chr6_957377-957398

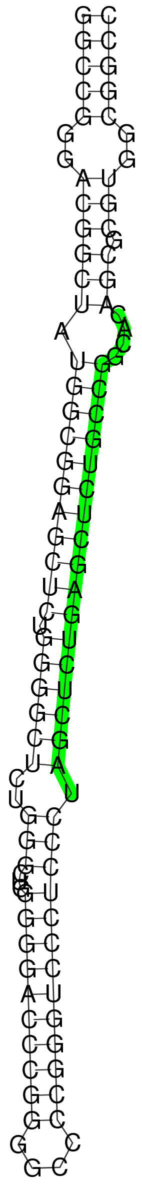

TAGCTCTGAGCTCTGCCGGCAC_chr2_6548022-6548043

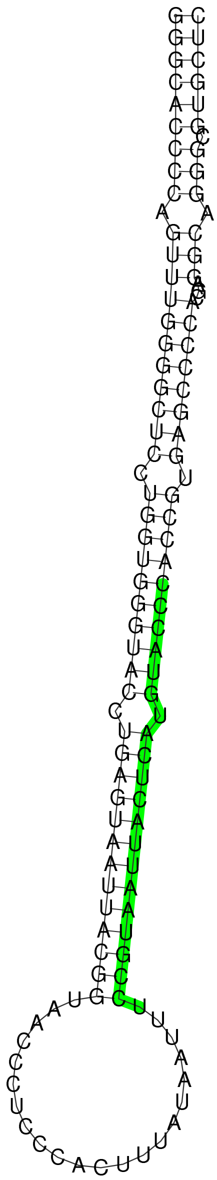

TCCGTAATTACTCATGTACCC_chr18_41651613-41651633

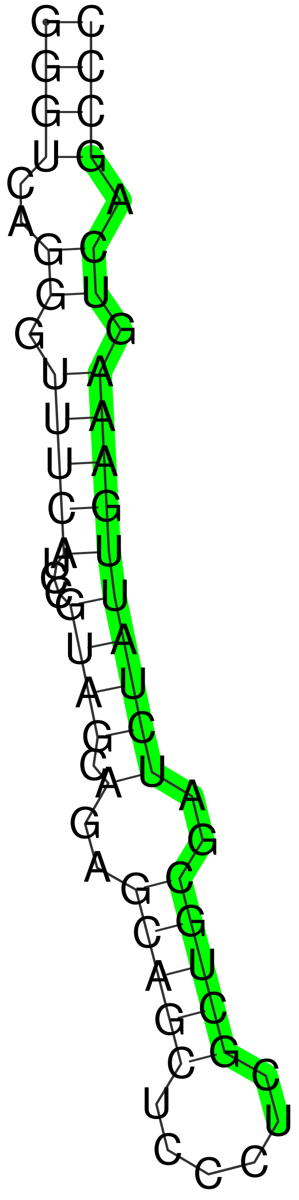

TCGCTGCGATCTATTGAAAGTCAG_chr5_93387252-93387275

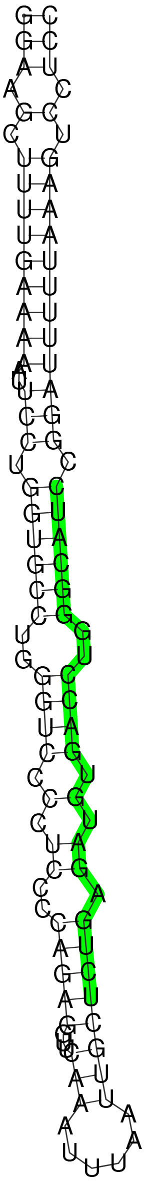

TCTGAGATGTGACCTGGGCATC_chr5_30185308-30185329

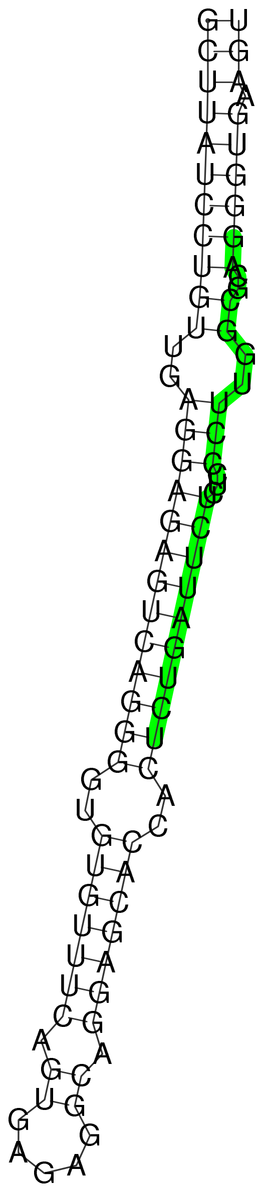

TCTGATTCTCTCCCTTGGCGCAG_chr14_86947273-86947295

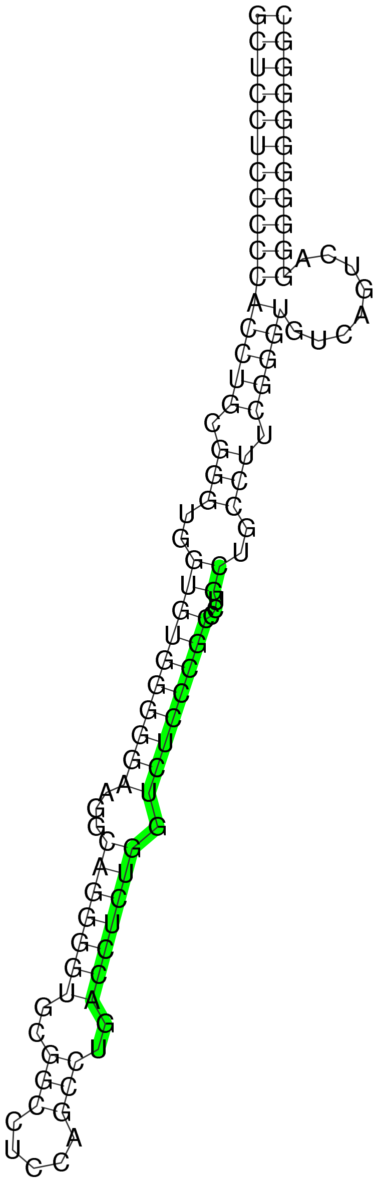

TGACCTCTGGTCTCCCGCCCTGC_chr6_956806-956828

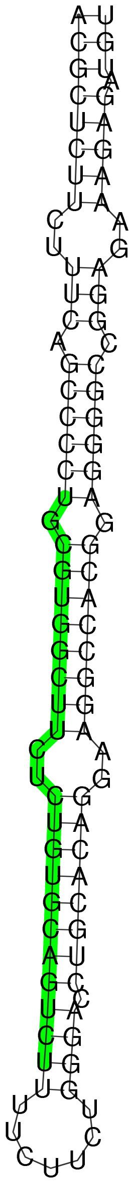

TGCGTGGCTTCTCTGTGCAGTCT_chr2_88902995-88903017

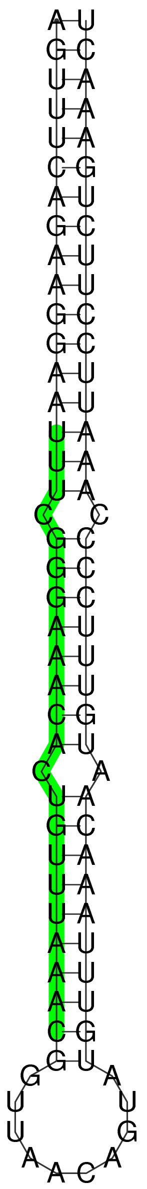

TTTCGGGAAACACTGTTTAAAC_chr7_15340262-15340283

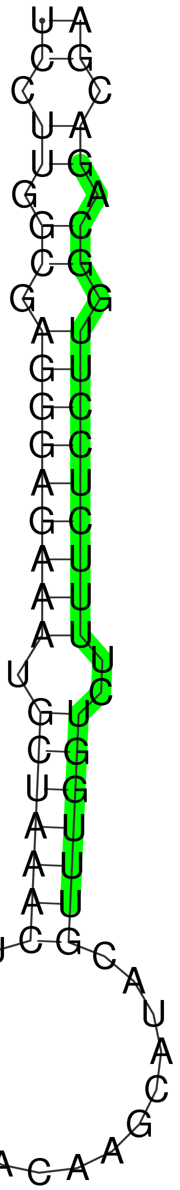

TTTGGTCTTTTCTCCTTGGCAG_chr12_49756097-49756118

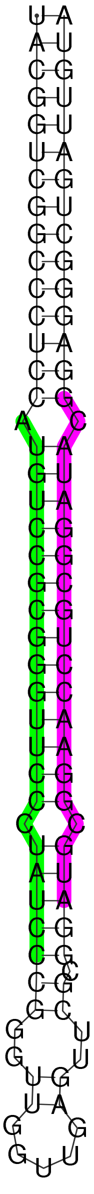

ATGTCCGCGGGTTCCCTATCC_chr5_5388521-5388541

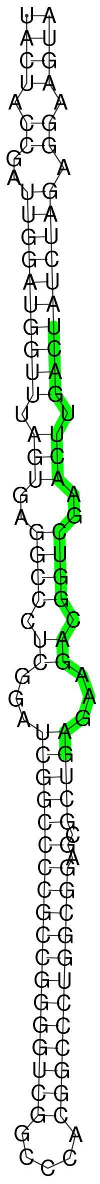

GAGAAGACGGTCGAACTTGACT_chr6_874231-874252

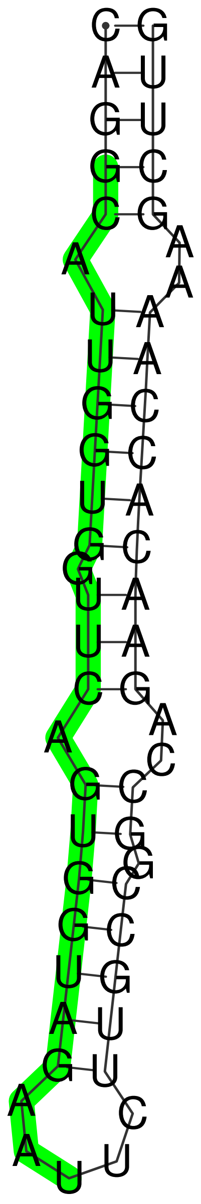

GCATTGGTGGTTCAGTGGTAGAAT_chr9_53318381-53318404

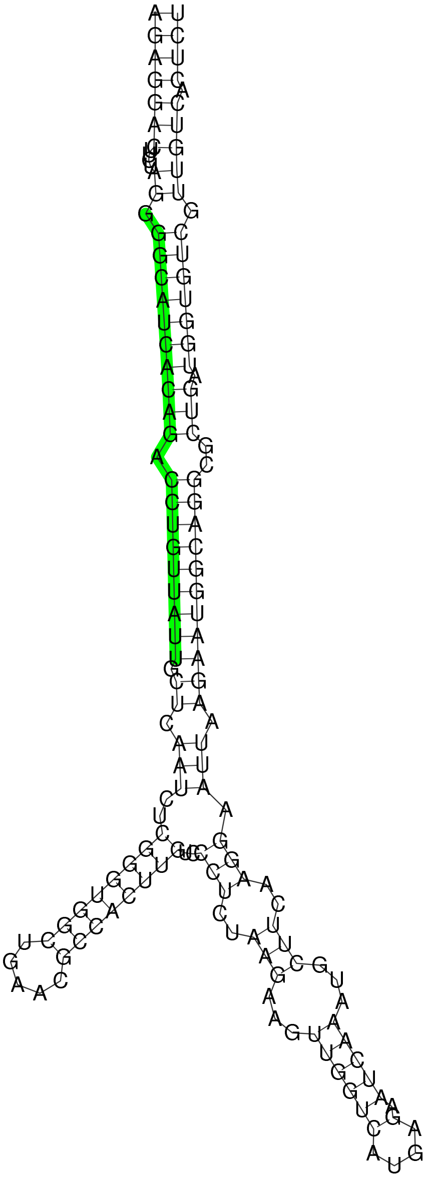

GGGCATCACAGACCTGTTATT_chr16_2672798-2672818

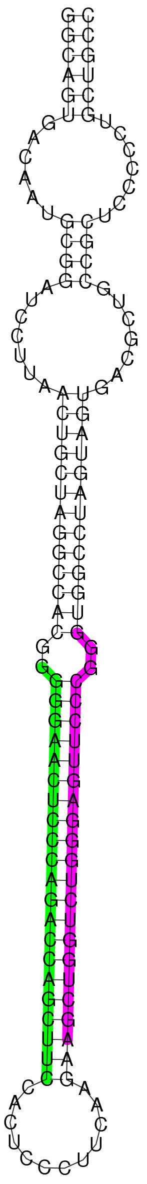

GGGGAACTCCCAGACCAGCTTC_chr3_10664434-10664455

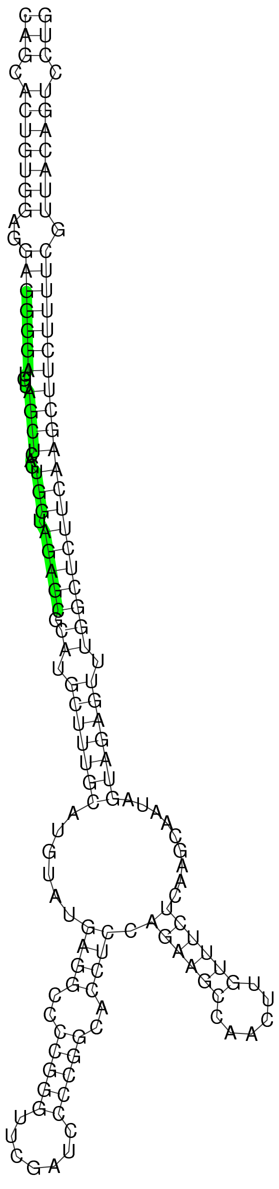

GGGGATGTAGCTCAGTGGTAGAGC_chr7_24380553-24380576

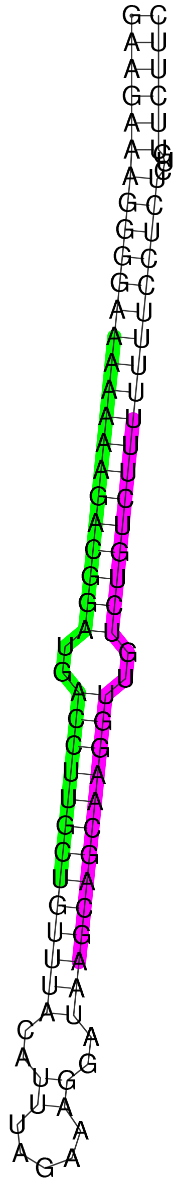

AAAAAAGACGGATGACCTTGCT_chr2_151102785-151102806

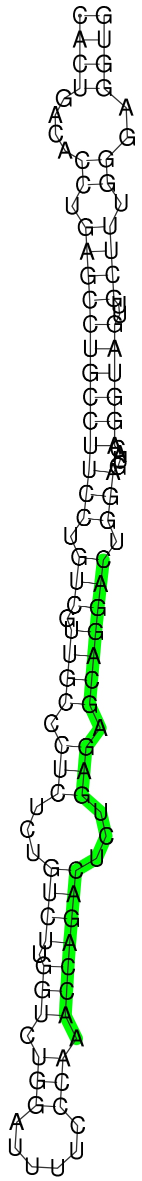

AACCAGACTCTGAGAGCAGGAC_chrX_8905463-8905484

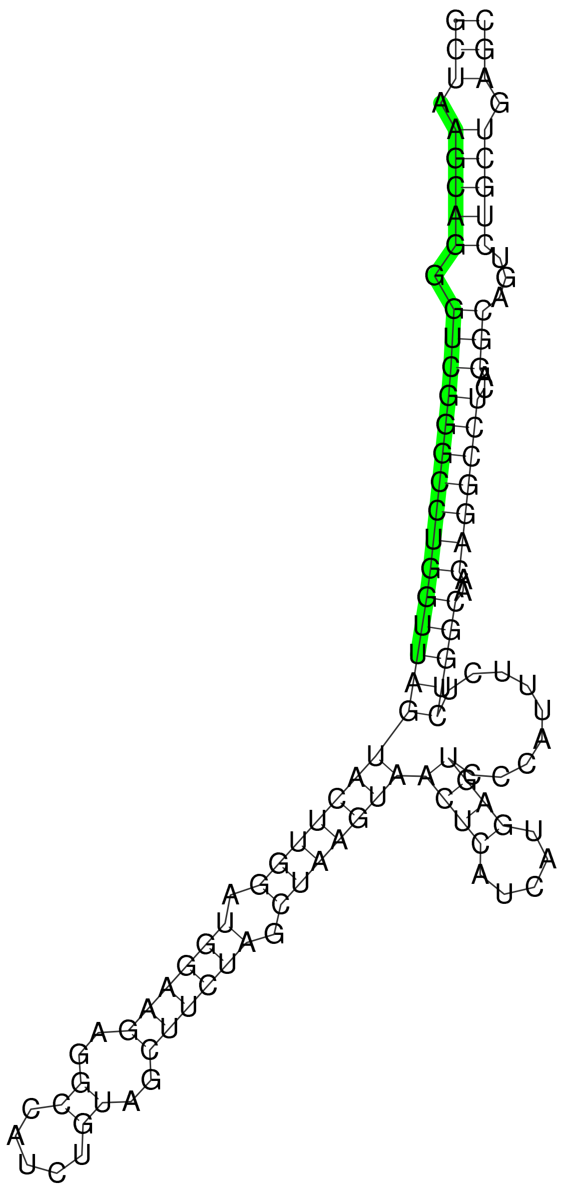

AAGCAGGGTCGGGCCTGGTT_chr14_114893858-114893877

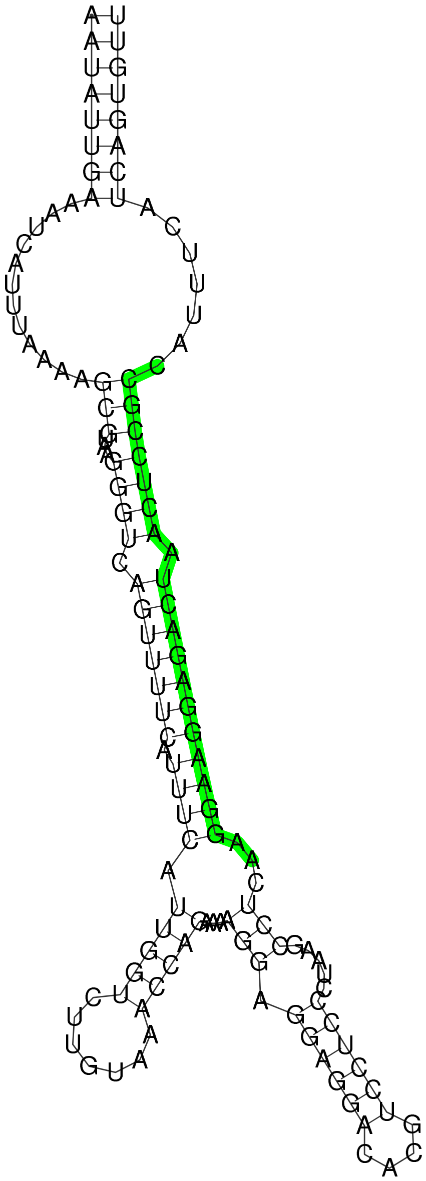

AAGGAAGGAGACTAACTCCGCC_chr15_146644014-146644035

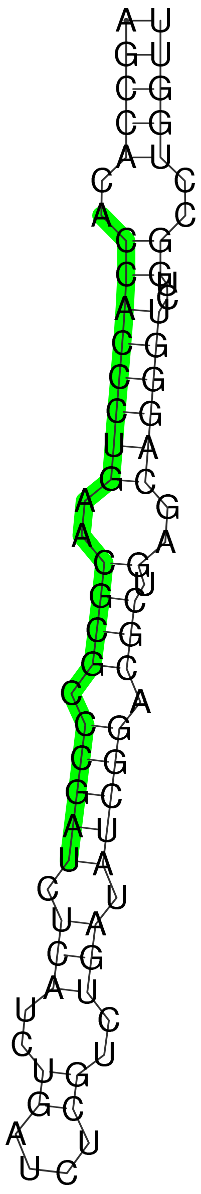

ACCACCCTGAACGCGCCCGAT_chr13_139351583-139351603

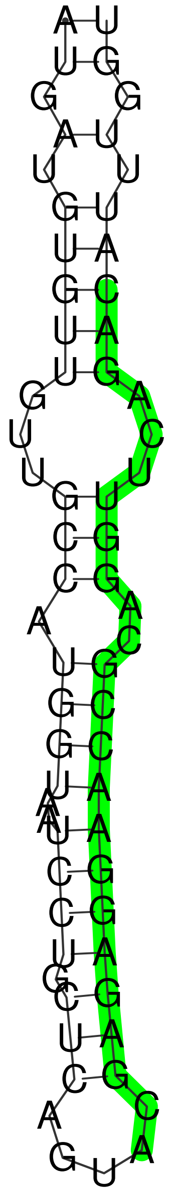

ACGAGAGGAACCGCAGGTTCAGAC_chr6_866912-866935

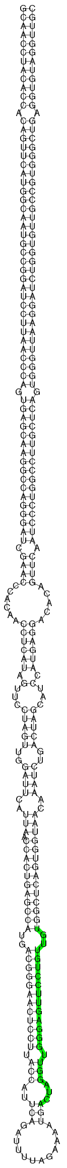

ACTAGGTTGGGAGTTCCTGTTGT_chr15_85376759-85376781

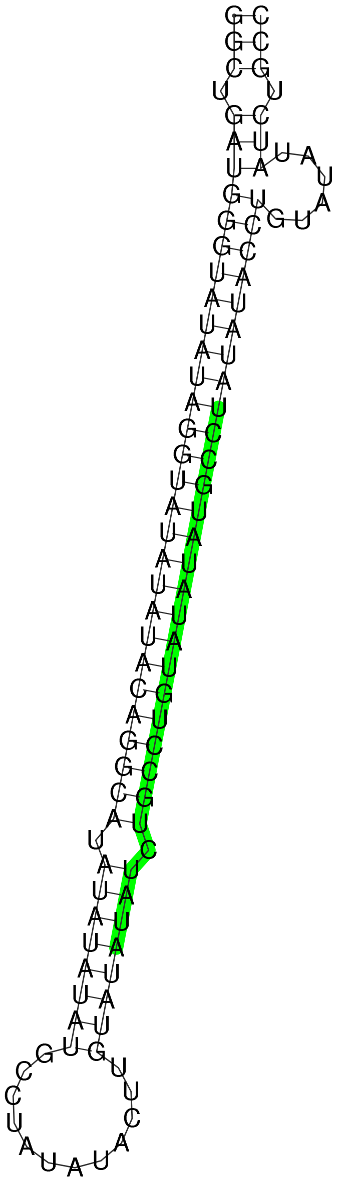

ATATCTGCCTGTATATATGCCT_chr13_136740234-136740255

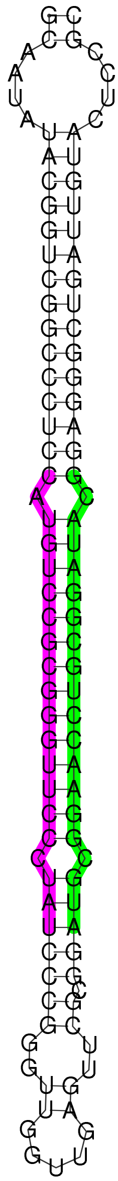

ATGCGGAACCTGCGGATACG_chr5_5388562-5388581

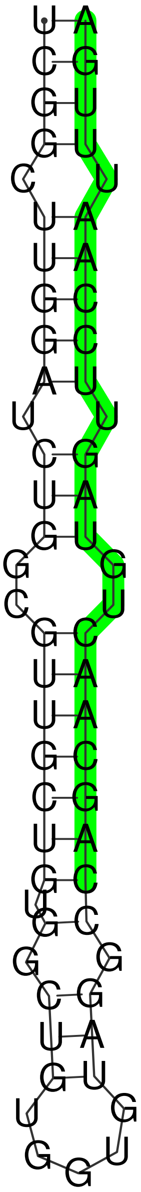

CAGCAACTGTAGTTCCAATTTGA_chr7_52432192-52432214

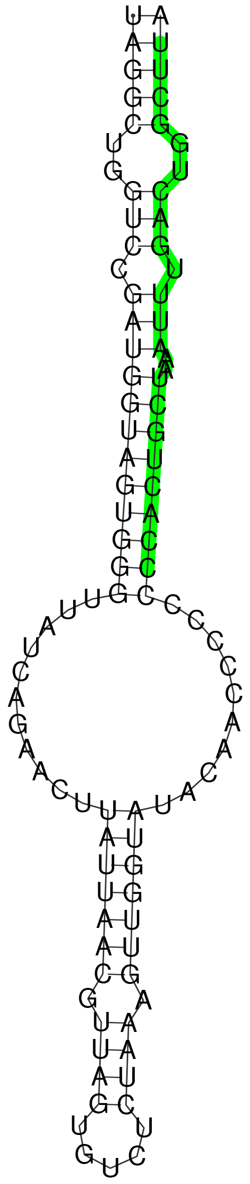

CCACTGCTAAATTTGACTGGCTT_chr10_6017532-6017554

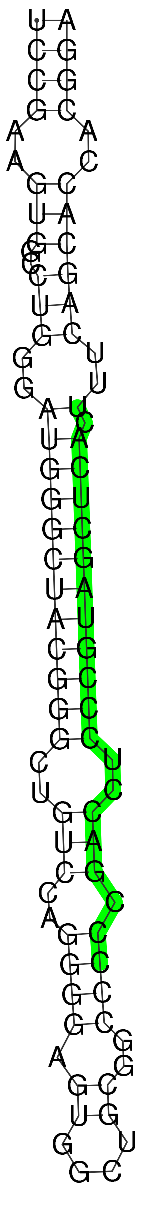

CCCGACCTCCCGTAGCTCACT_chr17_69264164-69264184

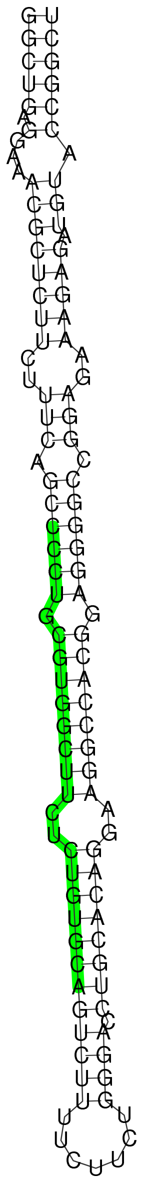

CCCTGCGTGGCTTCTCTGTGCA_chr2_88902992-88903013

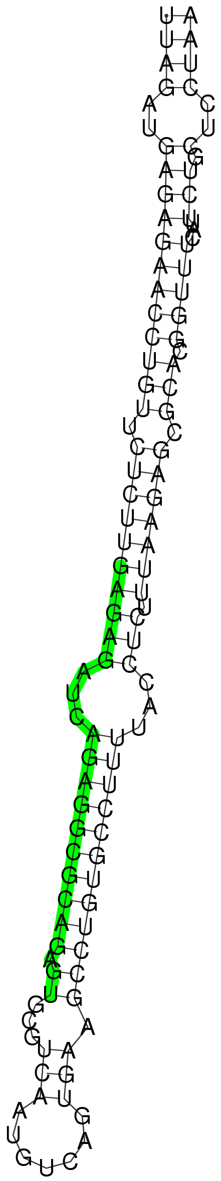

GAGAGATCAGAGGCGCAGAGT_chr13_149441301-149441321

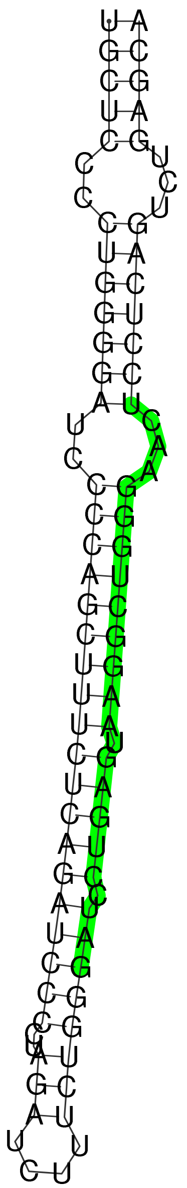

GATCCTGAGTAAGGCTGGGAACT_chr14_94928387-94928409

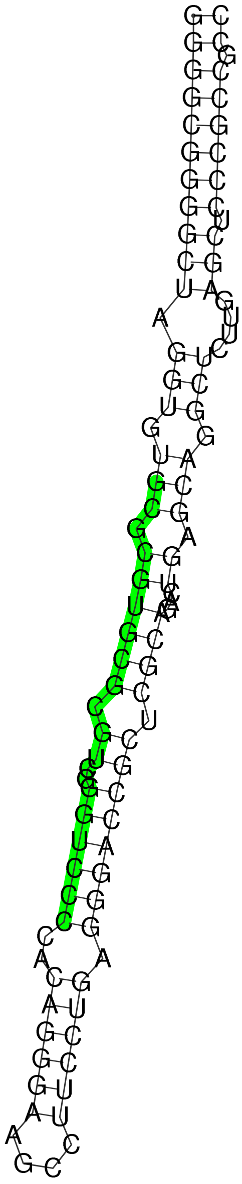

GCGCGTGCGCGTCGGGTCCC_chr2_140064440-140064459

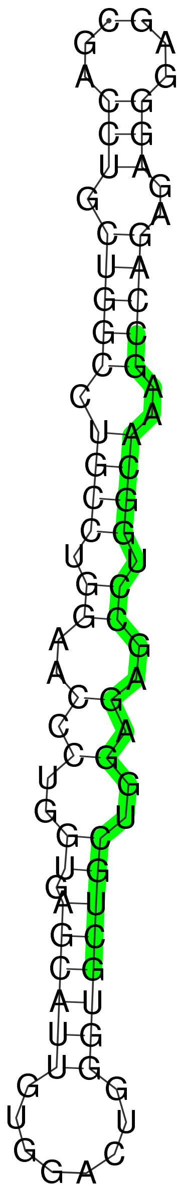

GCTGCTGGAGAGCCTGGCAAAGC_chr1_108195674-108195696

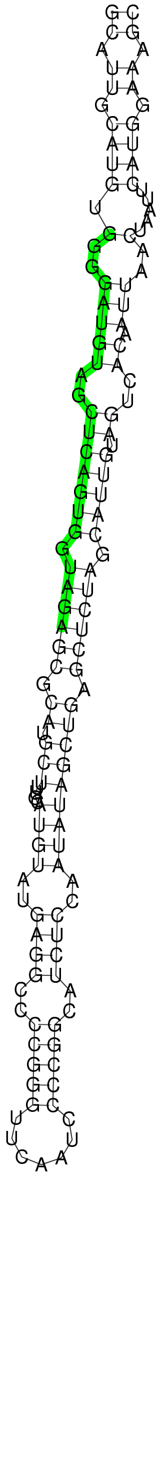

GGGGATGTAGCTCAGTGGTAGA_chr7_24295804-24295825

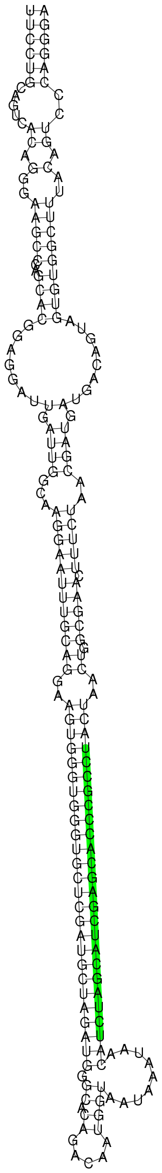

TCTAGCATCGAGCACCCGCCT_chr7_4037112-4037132

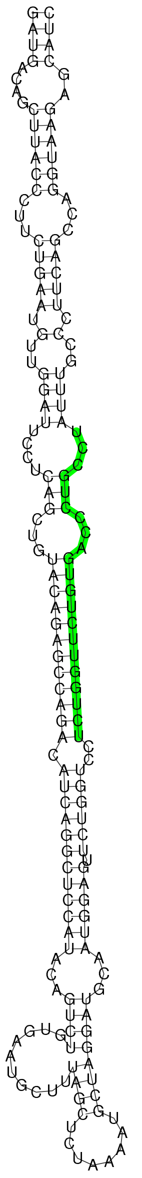

TCTGGTTCTGTGACCCTGCCT_chr7_55687381-55687401

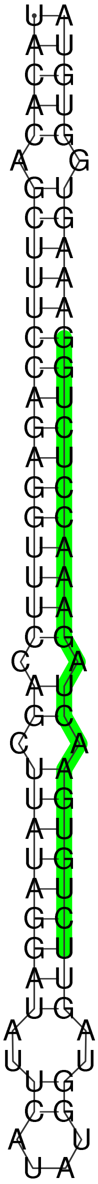

TCTGTGAACTAGAAACCTCTGG_chr8_122371914-122371935

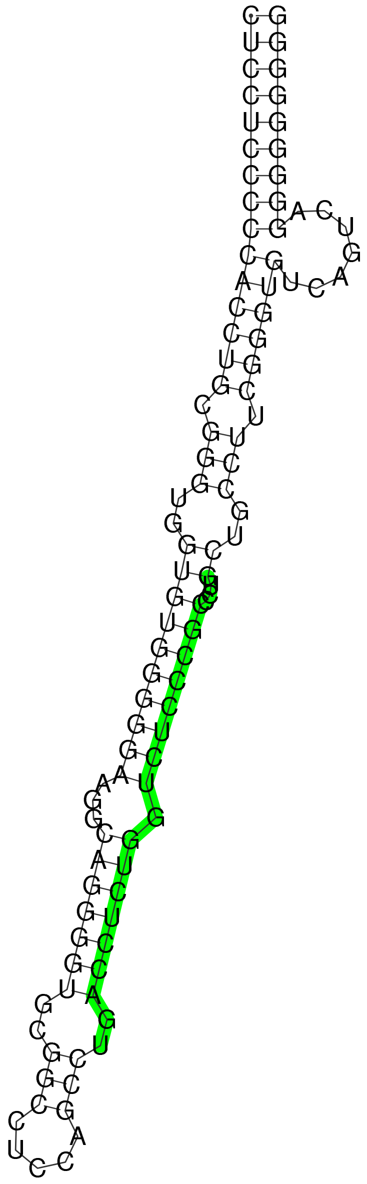

TGACCTCTGGTCTCCCGCCCTG_chr6_956806-956827

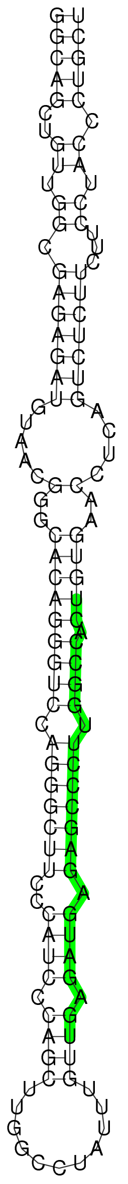

TGAGATGAGAGCCCTTGGCCACT_chr2_76694017-76694039

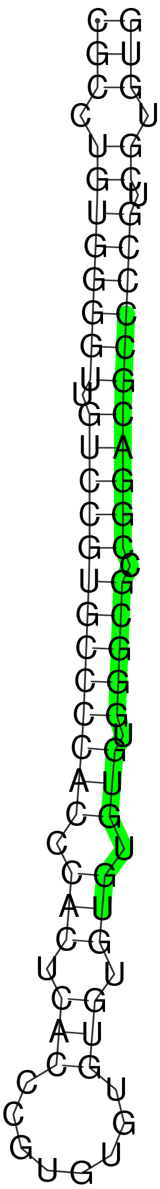

TGTGTGTGGGCGCCGGACGCC_chr6_872878-872898

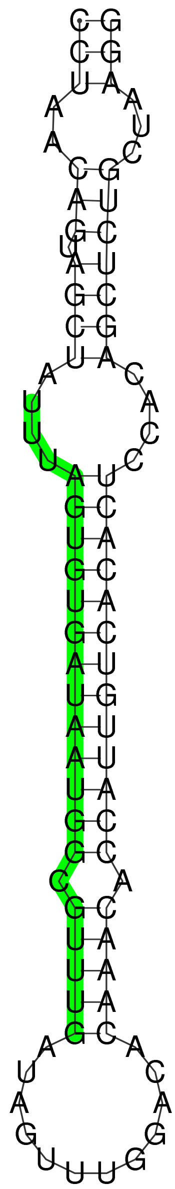

TTTAGTGTGATAATGGCGTTTG_chr1_179916351-179916372

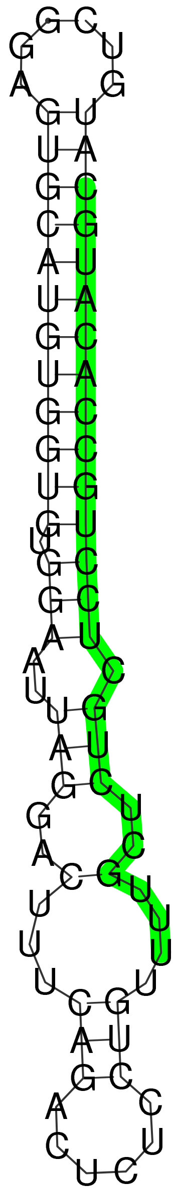

TTTGCTCTGCTCCTGCCACATGC_chr13_33474519-33474541

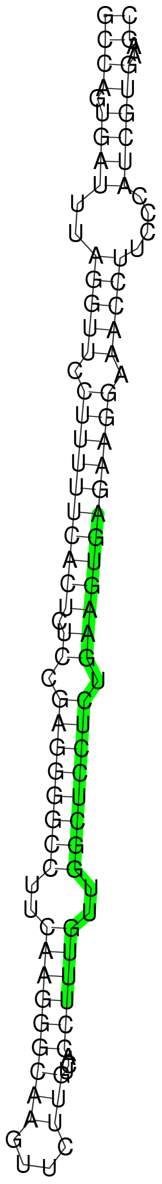

TTTGTTGGCTCCTCTGAAGTGA_chr2_7444711-7444732

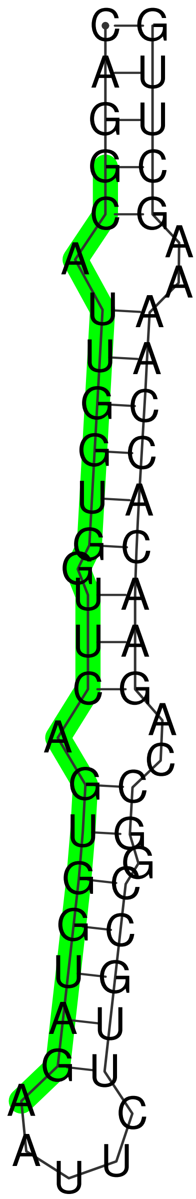

GCATTGGTGGTTCAGTGGTAGA_chr9_53318381-53318402

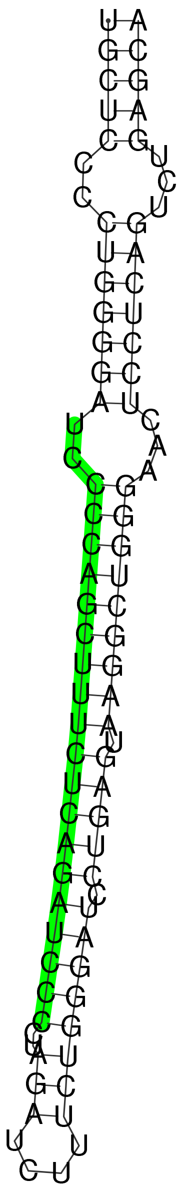

TCCCCAGCTTTCTCAGATCCC_chr14_94928352-94928372

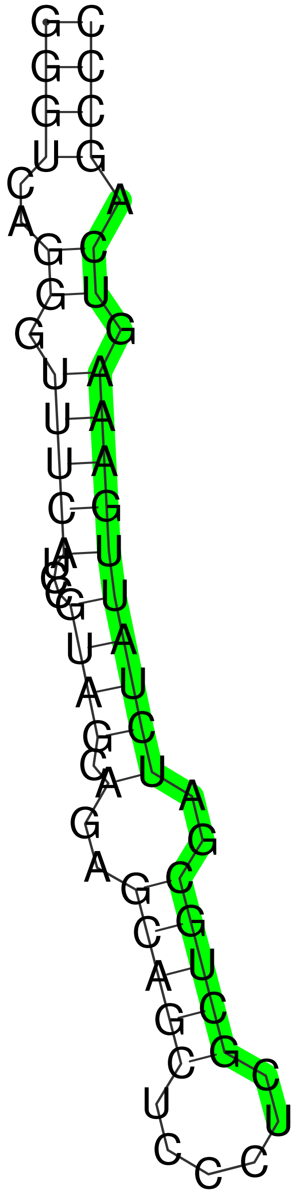

TCGCTGCGATCTATTGAAAGTCA_chr5_93387252-93387274
