# Supplementary material for: Identification of Genes Related to Growth and Lipid Deposition from Transcriptome Profiles of Pig Muscle Tissue
Source: PLoS One. 2015 Oct 27;10(10):e0141138. doi: 10.1371/journal.pone.0141138 (PMC4624711; doi:10.1371/journal.pone.0141138)
Supplement: S6 Fig — The vertical axis represents the expression value of miRNA in pig LD and the horizontal axis represents names of seven miRNAs. Error bars represent SE of expression. * *on the bars indicate significant differences (P<0.05) and *** indicate extremely significant differences (P<0.01) between DSP and YY breeds. DSP = Diannan Small-ear pig (n = 8). YY = Yorkshire (n = 8). (PDF) [file pone.0141138.s006.pdf]

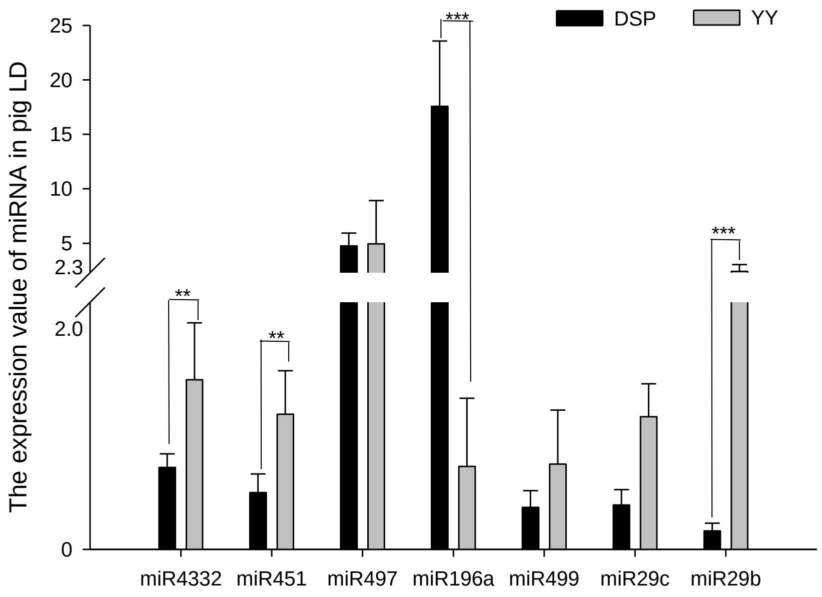

S6 Fig. The expression of seven miRNAs validated by qPCR in pig *longissimus dorsi* (LD) muscle tissue

Note: The vertical axis represents the expression value of miRNA in pig LD and the horizontal axis represents names of seven miRNAs. Error bars represent SE of expression. * *on the bars indicate significant differences (P<0.05) and *** indicate extremely significant differences (P<0.01) between DSP and YY breeds. DSP = Diannan Small Ear pig (n = 8). YY = Yorkshire (n = 8).
