# Supplementary material for: Identification of Genes Related to Growth and Lipid Deposition from Transcriptome Profiles of Pig Muscle Tissue
Source: PLoS One. 2015 Oct 27;10(10):e0141138. doi: 10.1371/journal.pone.0141138 (PMC4624711; doi:10.1371/journal.pone.0141138)
Supplement: S7 Fig — The pink circle represents the differential gene with the higher expression in the Diannan Small-ear pig-Tibetan pig (DSP-TP) group than that in the Landrace-Yorkshire (LL-YY) group, and the blue circle represents the differential gene with the lower expression. The red triangle represents differential miRNAs with higher expression in the DSP-TP group than that in the LL-YY group, and the green triangle represents the differential miRNAs with lower expression. (PDF) [file pone.0141138.s007.pdf]

S7 Fig. Network graphic of 18 differentially expressed miRNAs and 46 differentially expressed genes (DEGs) related to muscle growth.

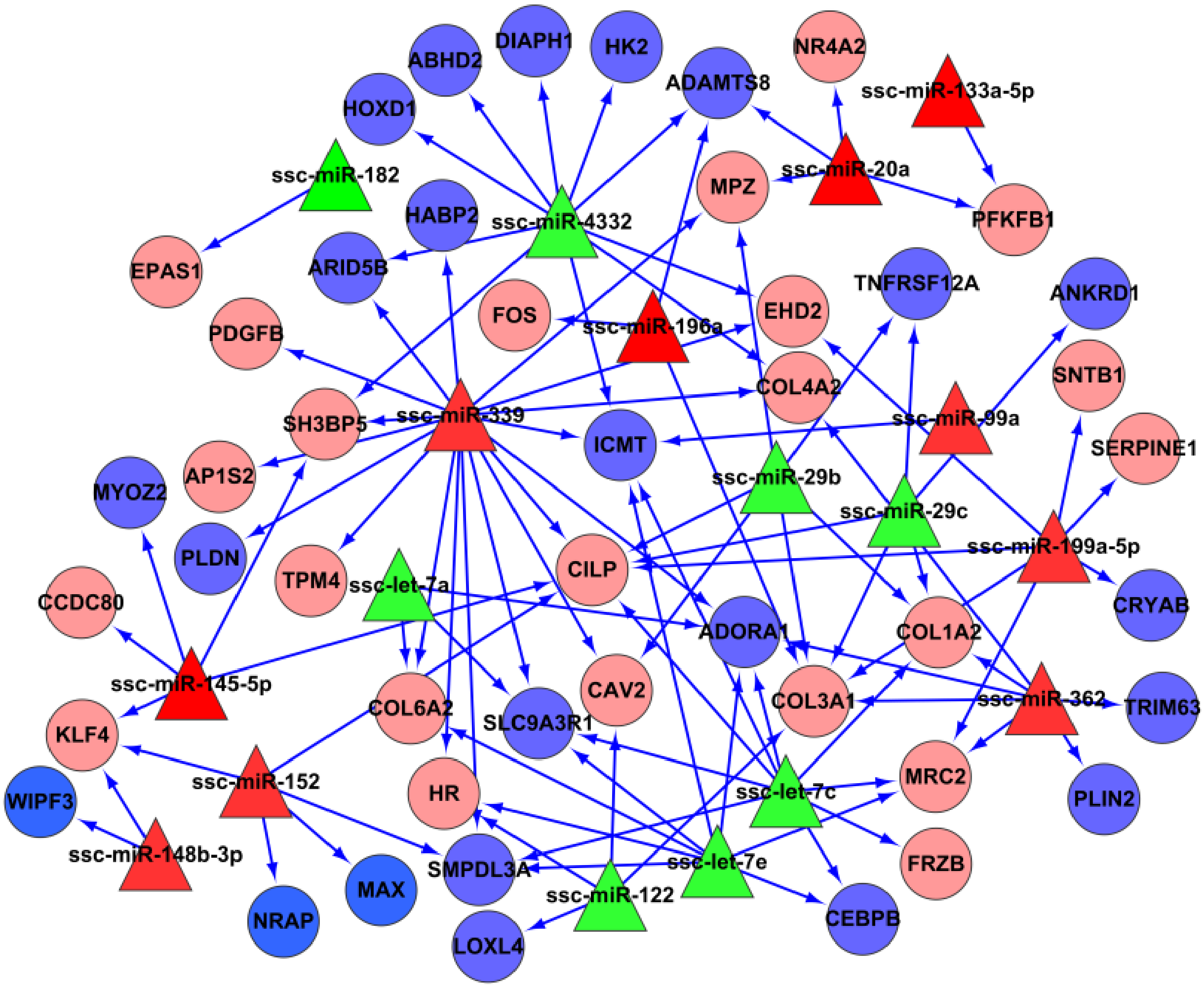

The pink circle represents the differential gene with the higher expression in the Diannan Small Ear pig-Tibetan pig (DSP-TP) group than that in the Landrace-Yorkshire (LL-YY) group, and the blue circle represents the differential gene with the lower expression. The red triangle represents differential miRNAs with higher expression in the DSP-TP group than that in the LL-YY group, and the green triangle represents the differential miRNAs with lower expression.
