# Supplementary material for: Identification of Genes Related to Growth and Lipid Deposition from Transcriptome Profiles of Pig Muscle Tissue
Source: PLoS One. 2015 Oct 27;10(10):e0141138. doi: 10.1371/journal.pone.0141138 (PMC4624711; doi:10.1371/journal.pone.0141138)
Supplement: S4 Table — (DOCX) [file pone.0141138.s012.docx]

**S4 Table. The sequencing yield of four libraries**

| **Items** | **DSP** | **TP** | **LL** | **YY** |
| --- | --- | --- | --- | --- |
| Number of reads | 5,587,108 | 6,194,657 | 3,642,041 | 8,358,625 |
| Number of trimmed reads | 5,068,711 | 5,043,036 | 2,975,233 | 7,087,919 |
| Number of annotated reads | 4,937,674 | 4,809,543 | 2,847,324 | 6,885,162 |
| Number of small RNAs | 67,433 | 69,565 | 85,558 | 62,977 |
| Number of annotated small RNAs | 24,280 | 27,097 | 28,069 | 24,681 |
| Number of annotated small RNAs mapped to miRBase (*Sus Scrofa*) | 208 | 247 | 258 | 237 |
| Number of small annotated RNAs mapped to Sus_scrofa.Sscrofa10.2.67.ncrna | 556 | 601 | 593 | 426 |
